# Targeted therapies in gynecological cancers: a comprehensive review of clinical evidence

**DOI:** 10.1038/s41392-020-0199-6

**Published:** 2020-07-29

**Authors:** Qiao Wang, Hongling Peng, Xiaorong Qi, Min Wu, Xia Zhao

**Affiliations:** 1 Department of Gynecology and Obstetrics, Development and Related Diseases of Women and Children Key Laboratory of Sichuan Province, Key Laboratory of Birth Defects and Related Diseases of Women and Children, Ministry of Education, West China Second Hospital, Sichuan University, Chengdu, Sichuan 610041 P.R. China; 2 Department of Biomedical Sciences, School of Medicine and Health Sciences, University of North Dakota, Grand Forks, ND 58203 USA

**Keywords:** Gynaecological cancer, Drug development

## Abstract

Advanced and recurrent gynecological cancers are associated with poor prognosis and lack of effective treatment. The developments of the molecular mechanisms on cancer progression provide insight into novel targeted therapies, which are emerging as groundbreaking and promising cancer treatment strategies. In gynecologic malignancies, potential therapeutic targeted agents include antiangiogenic agents, poly (ADP-ribose) polymerase (PARP) inhibitors, tumor-intrinsic signaling pathway inhibitors, selective estrogen receptor downregulators, and immune checkpoint inhibitors. In this article, we provide a comprehensive review of the clinical evidence of targeted agents in gynecological cancers and discuss the future implication.

## Introduction

Gynecological malignancies, mainly including ovarian, cervical, and endometrial cancer, seriously affect the health of women worldwide, contributing considerably to the global cancer burden. Epithelial ovarian cancer (OC) comprises ~90% of the malignant ovarian neoplasms, which is one of the leading causes of death in women.^1,2^ The 5-year overall survival (OS) rate of OC is ~47% for all stages, and >70% of patients are diagnosed at the advanced stage with an even lower 5-year OS rate.^3,4^ The standard-of-care fist-line treatments for OC are debulking surgery and perioperative platinum-based chemotherapy.^5,6^ Although the response rate of the first-line treatment is high, most of the patients will eventually experience relapses within the subsequent 3 years.^7^ At first relapse, ~20–25% of patients have platinum-resistant (disease recurs ≤6 months from the last platinum-based chemotherapy) or platinum-refractory (disease progress during or within 4 weeks of platinum-based chemotherapy) disease, with poor prognosis.^8,9^ In the platinum-resistant disease, single non-platinum agent is used, such as paclitaxel, docetaxel, pegylated liposomal doxorubicin (PLD), gemcitabine and topotecan. However, the response rates and outcomes are disappointing. Cervical cancer (CC), as the fourth most common female cancer globally, is also a major health problem especially for women in developing countries.^10^ High-risk human papilloma virus (HPV) infection is considered to be responsible for more than 90% of CC development.^11^ HPV overexpresses E6 and E7 oncoproteins which inhibit TP53 and RB1 proteins from altering cell cycle, apoptosis, and DNA repair.^12,13^ Thus, HPV testing is an important part of CC screening, and immunization against HPV (e.g., vaccines) has been designed to prevent CC.^14,15^ With early screening and effective treatments such as radical surgery or concurrent chemoradiation (a combination of radiation and chemotherapy), the cure rate of CC can reach 80% in the early-stage disease (FIGO stage I–II). The 5-year OS rate for all stages is ~66%. However, treatment options are limited and the survival rate is low for patients who present with distant metastatic disease, as well as those with unresectable recurrent disease and those who recur at distant. Endometrial cancer (EC), also known as uterine cancer, is the sixth most common female cancer.^10,16^ Elevated estrogen levels and increasing age are well-known risk factors of EC.^17,18^ Thus, the incidence of EC is increasing due to the increased life expectancy and obesity (causing elevated estrogen level). The standard treatment consists of surgery with or without adjuvant radiotherapy and/or chemotherapy, which is based on the risk of disease recurrence.^19^ Traditionally, EC has been classified in two types mainly according to histology and estrogen dependence. Furthermore, the Cancer Genome Atlas (TCGA) identified EC into four molecular subgroups: polymerase epsilon (POLE) ultramutated, microsatellite instability hypermutated, copy-number low, and copy-number high, each with a distinct prognosis.^20^ Most low-risk patients with early-stage disease can be cured by surgery and have good prognoses. However, the prognosis for advanced EC is poor with 5-year OS rate of 40–65% in stage III and 15–17% in stage IV disease, respectively.^21^ All those malignancies, when progressed to the advanced stage, have very poor prognoses under conventional treatment. Due to the lack of effective treatment for advanced-stage, refractory, recurrent, and drug-resistance disease, we are facing very tough challenges. However, based on the improved understanding of the mechanisms on cancer progression, targeted therapies are emerging as groundbreaking and promising treatment strategies.

In targeted therapies, individual patients are treated by agents targeting the changes in tumor cells that help them grow, divide, and spread. Currently in gynecological malignancies, potential therapeutic targets include tumor-intrinsic signaling pathways, angiogenesis, homologous-recombination deficiency (HDR), hormone receptors, and immunologic factors. The corresponding targeted agents include signaling pathway inhibitors, antiangiogenic agents, poly (ADP-ribose) polymerase (PARP) inhibitors, selective estrogen receptor downregulators, and Immune checkpoint inhibitors. For gynecological cancers, bevacizumab, olaparib, rucaparib, niraparib, and pembrolizumab have been approved by the US Food and Drug Administration (FDA) for selected patients with recurrent, metastatic, or high-risk diseases (Table 1). The clinical uses of these and other targeted agents are being actively and extensively investigated.

**Table 1:** FDA-approved targeted drugs for gynecological cancers

Target	Drug	Approval year	Indication		Administration
VEGFi	Bevacizumab (Avastin, Genentech)	2014	CC	Persistent, recurrent, or metastatic disease	15 mg/kg IV every 3 weeks with chemotherapy
		2014	OC	Platinum-resistant recurrent, and received no more than 2 prior chemotherapy regimens	10 mg/kg IV every 2 weeks with chemotherapy
		2016		Platinum-sensitive recurrent	15 mg/kg IV every 3 weeks with chemotherapy, and in maintenance
		2018		Advanced (FIGO stage III–IV)	
PARPi	Olaparib (Lynparza, AstraZeneca)	2014	OC	Advanced, with BRCAm, and have received three or more prior lines of chemotherapy	300 mg orally twice daily, until disease progression or unacceptable toxicity
		2017		Recurrent, and in complete or partial response to platinum-based chemotherapy	
		2018		Advanced, with BRCAm, and in complete or partial response to platinum-based chemotherapy	
	Rucaparib (Rubraca, Clovis)	2016	OC	Recurrent, with BRCAm, and have received two or more chemotherapies	600 mg orally twice daily, until disease progression or unacceptable toxicity
		2018		Recurrent and in a complete or partial response to platinum-based chemotherapy	
	Niraparib (Zejula, Tesaro)	2017	OC	Recurrent and in a complete or partial response to platinum-based chemotherapy	300 mg orally once daily, until disease progression or unacceptable toxicity
Anti-PD-1	Pembrolizumab (Keytruda, Merck)	2017	EC	Unresectable or metastatic, with a biomarker as MSI-H or dMMR	200 mg IV over 30 min every 3 weeks
		2018	CC	Recurrent or metastatic, with disease progression on or after chemotherapy, and expressing PD-L1	
Anti-PD-1 + VEGFi	Pembrolizumab (Keytruda, Merck) + lenvatinib (Lenvima, Eisai)	2019*	EC	Advanced disease without MSI-H/dMMR who have disease progression following prior systemic therapy, but are not candidates for surgery or radiation	Lenvatinib 20 mg orally once daily with pembrolizumab 200 mg IV over 30 min every 3 weeks

*CC* cervical cancer, *OC* epithelial ovarian, fallopian tube, or primary peritoneal cancer, *EC* endometrial cancer, *VEGFi* VEGF inhibitor, *PARPi* PARP inhibitor, *IV* intravenous infusion, *BRCAm* deleterious or suspected deleterious BRCA mutation, *MSI-H* microsatellite instability high, *dMMR* mismatch repair-deficient. *Accelerated approval

In this paper, we review the clinical efficacy and safety of the targeted therapies in gynecological cancers, by summarizing the results of previous clinical trials. We further describe the ongoing phase II/III clinical trials and expound future directions.

## Methods

A comprehensive literature review was performed on PubMed, including systematic reviews, review articles, clinical trials, and observation studies published in English. ClinicalTrials.gov was queried to collect the data of completed and ongoing clinical trials. For each approved targeted drug, the FDA website was searched for indication, usage and references as the basis for approval. Search terms included “gynecological cancers”, “ovarian cancer”, “cervical cancer”, “endometrial cancer”, “targeted therapy”, “antiangiogenic agents”, “PARP inhibitor”, “signaling pathway inhibitors”, “immune checkpoint inhibitors”, and each name of the targeted agent (e.g., “bevacizumab”, “olaparib”). We also used the ESMO and ASCO websites for preliminary results reported from ongoing trials.

### Antiangiogenic agents

Neovasculature is considered as a crucial process for tumor growth and progression.^22^ In decades, efforts have been made to develop vascular-targeted therapies for cancer treatment. Depending on the distinctly different mechanisms, vascular-targeted therapies include antiangiogenic agents and vascular-disrupting agents.^23^ Here, we focus on the action of antiangiogenic agents in this review.

**Fig. 1:**
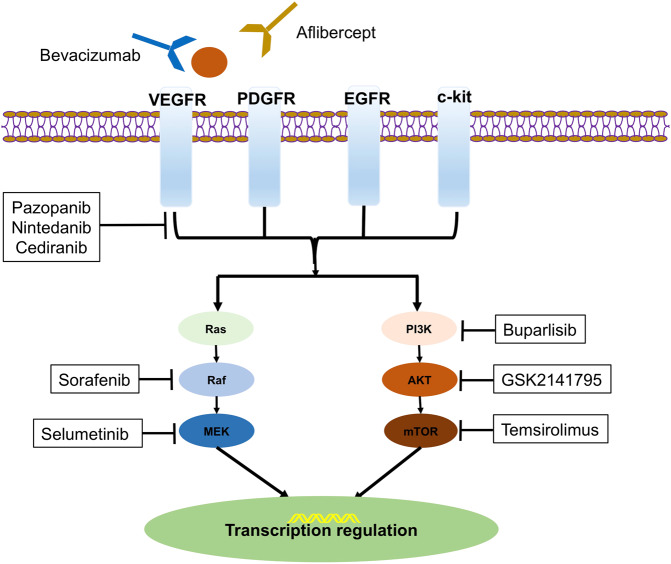
The VEGF, PI3K/AKT/mTOR, and Ras/Raf/MEK signal transduction pathway and therapeutic interventions. After ligand binding, the receptors initiate the signaling cascade reaction, which is overactive in cancer cells. The figure shows the main elements in those pathways and the therapeutic agents

Angiogenesis is a complex process regulated by various pro-angiogenic and antiangiogenic factors.^24^ Vascular endothelial growth factor (VEGF), a major driver of angiogenesis in solid tumors, binds to the VEGF receptors (VEGFR, including VEGFR-1/2/3) on target cells and initiates the signaling pathway through intracellular tyrosine kinases.^25^ It can initiate several endothelial cell signaling pathways and promote endothelial cell precursors from bone marrow.^24^ The VEGF pathway also interacts with the PI3K/AKT/mTOR pathway.^26,27^ Moreover, the process of angiogenesis is further modulated by the platelet-derived growth factor (PDGF) pathway, the fibroblast growth factor (FGF) pathway, the epidermal growth factor (EGF) pathway, and the angiopoietin family and their receptor tyrosine kinase (Tie2) pathways.^28^ There are complicated interplays of these pro-angiogenic pathways (Fig. 1).^29^ In addition, the VEGF expression can be induced by hypoxia-associated transcription factors, such as hypoxia inducible factors (HIF1A and HIF2A). It is also associated with other genetic alterations such as TP53, RAS, and EGFR.^30^

In tumor cells, the expression levels of the pro-angiogenic factors, especially VEGF, are upregulated to develop tumor’s own endogenous blood vessels, which is associated with the poor prognosis.^22,31^ Therefore, antiangiogenic therapies are developed by inhibiting target signaling pathways at different points. The main classes of antiangiogenic agents are anti-VEGF monoclonal antibodies (e.g., bevacizumab), soluble VEGFRs (e.g., aflibercept), inhibitors of angiopoietin-Tie2 receptor (e.g., trebananib), and tyrosine kinase inhibitors (e.g., cediranib).^24,32^ Tyrosine kinases are enzymes that catalyze the transfer of phosphate from adenosine triphosphate (ATP) onto target proteins to elicit a response.^33^ Tyrosine kinase inhibitors (TKIs) are small molecules which can block intracellular tyrosine kinases in multiple signaling pathways (e.g., VEGF, EGF).

A number of antiangiogenic agents, such as bevacizumab, pazopanib, sunitinib, sorafenib, vandetanib, aflibercept, axitinib, regorafenib, ramucirumab, and lenvatinib are FDA-approved for cancer treatment (e.g., colorectal cancer, lung cancer, renal cell carcinoma, and thyroid cancer). For gynecological cancers, bevacizumab was the first and only FDA-approved anti-VEGF drug. As of January 2020, there are a dozen of completed phase III trials assessing the efficacy and safety of antiangiogenetic agents for gynecological cancers, especially in OC. The main data from completed Phase II/III clinical trials are summarized in Tables 2 and 3.

**Table 2:** Completed phase III trials of antiangiogenic agents in gynecological cancers

ID	Cancer/condition	No.	Intervention	mPFS (mon.)	mOS (mon.)	SAEs (%)	Refs
NCT00483782 ICON7	OC/high-risk stage I–IIa, IIb–IV	1528	(1) PC	17.5	58.6	–	^37^
			(2) PC + bevacizumab	19.9, *P* = 0.25	58.0, *P* = 0.85	–	
NCT00976911 AURELIA	OC/platinum-resistant recurrent	361	(1) Single-agent chemotherapy	3.4	13.3	27.1	^42^
			(2) Chemotherapy + bevacizumab	6.7, *P* < 0.001	16.6, *P* = 0.174	31.28	
NCT00434642 OCEANS	OC/platinum-sensitive recurrent	484	(1) GC + placebo	8.4	32.9	25.32	^40^
			(2) GC + bevacizumab	12.4, *P* < 0.0001	33.6, *P* = 0.65	36.44	
NCT00262847 GOG-0218	OC/stage III–IV	1873	(1) PC + placebo	10.3	41.1	38.49	^35^
			(2) PC + bevacizumab throughout	14.1, *P* < 0.001	40.8, *P* = 0.34	41.19	
			(3) PC + bevacizumab combination only	11.2, *P* = 0.16	43.4, *P* = 0.53	46.37	
NCT00565851 GOG-0213	OC/platinum-sensitive recurrent	674	(1) PC	10.4	37.3	86	^41^
			(2) PC + bevacizumab	13.8, *P* < 0·0001	42.2, *P* = 0.045	96	
NCT00803062 GOG-0240	CC/metastatic, persistent, or recurrent	452	(1) PC	6	13.3	37.5	^42,43^
			(2) PT			34.58	
			(3) PC + bevacizumab			47.75	
			(4) PT + bevacizumab	8.2, *P* = 0.002	16.8, *P* = 0.007	55.96	
NCT00532194 ICON6	OC/platinum-sensitive recurrent	486	(1) Chemotherapy + placebo	8.7	–	–	^73^
			(2) Chemotherapy + cediranib throughout	9.9		–	
			(3) Chemotherapy + cediranib combination only	11, *P* < 0.0001		–	
NCT01015118 AGO-OVAR12	OC/stage IIb–IV	1503	(1) PC + placebo	16.6	62.8	34.89	^67^
			(2) PC + nintedanib	17.2, *P* = 0.24	62, *P* = 0.087	42.02	
NCT00866697 AGO-OVR16	OC/stage II–IV, after first-line chemotherapy	940	(1) Placebo	12.3	64.0	11.06	^63^
			(2) Pazopanib	17.9, *P* = 0.0021	59.1, *P* = 0.64	25.37	
NCT01204749 TRINOVA-1	OC/recurrent	919	(1) Paclitaxel + placebo	5.4	17.3	52	^78^
			(2) Paclitaxel + trebananib	7.2, *P* < 0.0001	19.0, *P* = 0.19	53	
NCT01281254 TRINOVA-2	OC/recurrent	223	(1) PLD + placebo	7.2	17.0	72	^81^
			(2) PLD + trebananib	7.6, *P* = 0.57	19.4, *P* = 0.76	73	
NCT01493505 TRINOVA-3	OC/stage III–IV	1164	(1) PC + placebo	15.0	–	66	^80^
			(2) PC + trebananib	15.9, *P* = 0.36		73	

*ID* identifier, *No.* enrollment number, *mPFS* median progression-free survival, *mOS* median overall survival, *Mon.* months, *SAEs* serious adverse events, *Refs* references, *Stage* FIGO stage, *PC* paclitaxel + carboplatin, *GC* gemcitabine + carboplatin, *PT* topotecan + paclitaxel, *PLD* pegylated liposomal doxorubicin

**Table 3:** Completed phase II trials of antiangiogenic agents in gynecological cancers

ID	Cancer/condition	No.	Intervention	ORR (%)	mPFS (mon.)	mOS (mon.)	SAEs (%)	Refs
NCT00025233	CC/persistent or recurrent	46	Bevacizumab	10.9	3.4	7.29	58.7	^45^
NCT00548418 GSK107278	CC/persistent or recurrent	27	Bevacizumab + topotecan + cisplatin	59	7.1	13.2	44.44	^46^
NCT00369122 RTOG0417	CC/stage Ib–IIIb	60	Bevacizumab + cisplatin + radiotherapy	68.7	–	–	22.03	^49^
–	CC/advanced or recurrent	34	Bevacizumab + PC	88	9	26	–	^47^
NCT00937560 OCTAVIA	OC/stage IIb–IV	189	Bevacizumab + PC	84.6	23.7	–	22.8	^396^
NCT01010126	EC/stage III–IV	26	Bevacizumab + temsirolimus	25.1	6.0	11.5	61.5	^60,339^
	OC/stage III–IV	58		6.4	5.6	16.3	58.6	
NCT01305213 GOG-0186I	OC/recurrent	107	(1) Bevacizumab	28.2	4.8	–	16.98	^397^
			(2) Bevacizumab + fosbretabulin	35.7	7.3, *P* = 0.05		29.6	
NCT00696670	OC/resistant	39	Bevacizumab + erlotinib	23.1	4	–	30	^398^
NCT00945139	OC/platinum-resistant recurrent	46	Bevacizumab + PLD	30.2	6.6	33.2	6.52	^399^
NCT01091259	OC/recurrent	29	Bevacizumab + irinotecan	27.6	6.8	15.4	31	^400^
NCT00886691 GOG-0186G	OC/recurrent	150	(1) Bevacizumab	12.1	4.5	17.3	32	^401^
			(2) Bevacizumab + temsirolimus	22.2	5.9, *P* = 0.39	16.6, *P* = 0.55	46.7	
NCT00407563 ACORN	OC/platinum-resistant recurrent	48	Bevacizumab + abraxane	50	8.08	17.15	27.1	^402^
NCT00267696 OSU-05070	OC/platinum-resistant recurrent	45	Bevacizumab + GC	69	13.3	36.1	8.9	^403^
NCT00977574 GOG-0086P	EC/stage III–IV	339	(1) Bevacizumab + PC	60	–	34	42.9	^404^
			(2) Temsirolimus + PC	55		25	50.4	
			(3) Bevacizumab + carboplatin	53		25.2	46.5	
NCT01770171 MITO END-2	EC/advanced or recurrent	108	(1) PC	53.1	10.5	29.7	–	^54^
			(2) PC + bevacizumab	74.4	13.7, *P* = 0.43	40.0, *P* = 0.24		
NCT01005329 RTOG 0921	EC/high risk	34	Bevacizumab + cisplatin + radiotherapy	The 2-year estimate of OS was 96.7%			26.7	^53^
NCT00879359	EC/advanced or recurrent	15	Bevacizumab + PC	73	18	58	73.3	^52^
NCT00723255 GOG-0229G	EC/recurrent	43	Bevacizumab + temsirolimus	24.5	5.6	16.9	63.3	^405^
NCT00301964 GOG-0229E	EC/persistent or recurrent	56	Bevacizumab	13.5	4.2	10.5	34.6	^51^
-	EC/persistent or recurrent	46	Bevacizumab + pemetrexed	41	7.9	25.7	52	^406^
NCT01468909	OC/recurrent	106	(1) Paclitaxel	31.8	7.5	23.3	30.00	^407^
			(2) Pazopanib + paclitaxel	22.7	6.2, *P* = 0.20	20.7, *P* = 0.90	42.31	
NCT01644825 MITO-11	OC/stage Ic–IV	74	(1) Paclitaxel	25	6.5	–	34	^408^
			(2) Pazopanib + paclitaxel	56	16.1, *P* <0.01		46	
NCT00430781	CC/stage IVb, persistent, or recurrent	230	(1) Pazopanib	9	4.22	–	37.84	^257^
			(2) Lapatinib	5	3.99, *P* = 0.013		29	
NCT02055690	OC/recurrent	21	(1) Pazopanib	22	3.7	–	–	^45^
			(2) Pazopanib + fosbretabulin	18	7.6, *P* = 0.08			
NCT01669798	OC/recurrent, bevacizumab-resistant	27	Nintedanib	7.4	1.8	16	22.2	^68^
NCT01225887 GOG-0229K	EC/recurrent	37	Nintedanib	9.4	3.3	10.1	43.8	^69^
NCT01210222 GOG-0229L	EC/recurrent	35	Trebananib	3.1	1.7	6.6	43	^82^
NCT01253681	OC/recurrent	61	(1) Placebo	27	4.6	–	64	^409^
			(2) Trebananib	19	5.7		55	
			(3) Trebananib + paclitaxel	37	7.2		65	
NCT01111461	EC/recurrent	133	Lenvatinib	14.3	5.4	10.6	46.62	^410^
NCT00278343	OC/recurrent	74	Cediranib	26	4.9	18.9	6.8	^72^
NCT01132820 GOG-0229J	EC/recurrent	48	Cediranib	12.5	3.65	12.5	41.7	^74^
NCT00888173 GOG-0229I	EC/recurrent	43	Brivanib	7	3.3	10.7	41.86	^95^
NCT01267253 GOG-0227G	CC/recurrent	28	Brivanib	8	3.2	7.9	50	^94^
NCT02867956	OC/platinum-refractory	35	Apatinib + etoposide	54	–	–	5.7	^87^
NCT02867956	OC/recurrent	29	Apatinib	41.4	5.1	14.5	31	^86^
NCT00979992 GOG-0254	OC/clear cell, recurrent or persistent	30	Sunitinib	6.7	2.7	12.8	–	^91^
NCT00388037	OC/recurrent	30	Sunitinib	3.3	4.1	–	50.00	^90^
NCT00543049 AGO 2.11	OC/platinum-resistant recurrent	76	Sunitinib (noncontinuous/continuous)	16.7/5.4	4.8/4.9	13.6/13.7	–	^89^
NCT00768144	OC/recurrent, platinum-refractory	35	Sunitinib	8.3	9.9	–	19.44	^88^
NCT00478426	EC/metastatic or recurrent	33	Sunitinib	18.1	3	19.4	52	^92^
NCT00389974	CC/advance or metastatic	19	Sunitinib	0	3.5	–	73.68	^93^

*ORR* objective response rate

### Bevacizumab

Bevacizumab is a humanized anti-VEGF monoclonal antibody, which is the best-known antiangiogenetic agent. In gynecological cancers, bevacizumab is currently approved by FDA as combination treatment and/or maintenance treatment for selected patients with: (1) persistent, recurrent, or metastatic CC; (2) advanced or recurrent OC (including stage III/IV epithelial ovarian cancer, fallopian tube, or primary peritoneal cancer) (Table 1). The decisions of these indications are mainly grounded on findings from the following six Phase III clinical trials (five for OC and one for CC) (Table 2).

GOG-0218 trial (NCT00262847) evaluated the efficacy of bevacizumab (15 mg/kg intravenously every 3 weeks) in combination with chemotherapy plus/without bevacizumab maintenance for patients with newly diagnosed advanced OC following initial surgery. The median progression-free survival (PFS) was increased in the bevacizumab-concurrent plus maintenance arm when compared with control (chemotherapy alone) arm (3.8 months longer, *P* < 0.001). PFS was not significantly increased in the bevacizumab-concurrent arm (without bevacizumab maintenance).^34^ However, final results of this trial were updated in July, 2019. When compared with the control arm, there is no significant increase in the median OS either in the bevacizumab-concurrent plus maintenance arm or in the bevacizumab-concurrent arm. In a subset analysis stratified by stage, for patients with stage IV disease, the control and bevacizumab-concurrent arms were associated with a median OS of 32.6 and 34.5 months, respectively. The median OS was increased in patients with stage IV disease who received bevacizumab-concurrent plus maintenance (42.8 months, HR, 0.75; 95% CI, 0.59–0.95).^35^ Another phase III trial, ICON7 (NCT00483782) found a modest increase in the median PFS (2.4 months longer, *P* = 0.25) with no OS benefit in chemotherapy plus bevacizumab (both concurrence and maintenance) arm in the updated analyses.^36^ However, in a subset analysis of patients at high risk of progression, a significant difference in the median OS was noted between patients in chemotherapy plus bevacizumab arm and those in chemotherapy alone arm (39.3 vs. 34.5 months, *P* = 0·03).^37^ Data from these two trials did not show a statistically different quality of life (QOL) in the whole study population.^38^ Owing to the above trials, the FDA approved bevacizumab in combination with chemotherapy and followed as maintenance therapy for newly diagnosed advanced OC patients after initial surgical resection.

For patients with platinum-sensitive recurrent OC, OCEANS trial (NCT00434642) showed that the median PFS was significantly increased (4 months longer, *P* < 0.0001) in chemotherapy plus bevacizumab arm compared with chemotherapy alone.^39^ However, no significant difference in OS was observed at the final analysis.^40^ On the other hand, another phase III trial GOG-0213 (NCT00565851) showed that the addition of bevacizumab to chemotherapy led to a significant difference in both median PFS (3.4 months longer, *P* < 0.0001) and OS (4.9 months longer, adjusted *P* = 0.0447) in patients with platinum-sensitive recurrent OC.^41^ The FDA approved bevacizumab in combination with first-line chemotherapy and followed as maintenance therapy for platinum-sensitive recurrent OC patients in 2016.

For patients with platinum-resistant recurrent OC, an open-label phase III trial, AURELIA (NCT00976911), found that the addition of bevacizumab to chemotherapy improved the median PFS (3.3 months longer, *P* < 0.001), but with no benefit in OS at the final analysis.^42,43^ Based on this trail, the FDA approved bevacizumab in combination with chemotherapy for platinum-resistant recurrent OC patients who received no more than two prior chemotherapy regimens.

Another phase III trial (NCT01081262), studying different chemotherapy regimens with or without bevacizumab as the first-line therapy in treating patients with mucinous epithelial OC, was closed early due to slow accrual.^44^ An ongoing phase III trial (NCT03635489) is evaluating the efficacy and safety of bevacizumab plus chemotherapy in Chinese participants with newly diagnosed advanced OC.

For CC, phase II trials (e.g., NCT00548418) demonstrated that the combination of chemotherapy and bevacizumab in patients with recurrent or persistent CC had an objective response rate (ORR) of 59–88%.^45–47^ Furthermore, a phase III trial, GOG-0240 (NCT00803062), revealed an improvement in the median PFS (2.2 months longer, *P* = 0·0002) and OS (3.5 months longer, *P* = 0.007) among patients receiving chemotherapy plus bevacizumab compared with those receiving chemotherapy alone.^48^ Based on this trail, the FDA approved bevacizumab in combination with standard chemotherapy for metastatic, persistent, or recurrent CC. For locally advanced CC, a phase II trial (NCT00369122) showed concurrent cisplatin-based chemoradiotherapy and bevacizumab had an ORR of 68.7%.^49^ Another phase II/III trial (JCOG1311) has been initiated to compare different chemotherapy regimens with or without bevacizumab in stage IVb, recurrent or persistent CC.^50^

Currently, there are limited results of phase III studies assessing the efficacy of bevacizumab for patients with EC. In a phase II trial (NCT00301964) for persistent or recurrent EC, the single-agent bevacizumab therapy was shown to have an ORR of 13.5%, with the median PFS and OS being 4.2 and 10.5 months, respectively.^51^ Another phase II trial (NCT00879359) for advanced or recurrent EC showed that bevacizumab in combination with chemotherapy had an ORR of 73%, presenting a median PFS of 18 months and a median OS of 58 months.^52^ For patients with high-risk EC, postoperative bevacizumab added to chemotherapy and pelvic radiotherapy resulted in a high OS rate (at 2 years) of 96.7% and a disease-free survival rate of 79.1%, which was reported in a phase II trial (NCT01005329).^53^ However, bevacizumab plus chemotherapy failed to demonstrate a significant increase in PFS of patients with advanced or recurrent EC, reported by the MITO END-2 trial (NCT01770171) in 2019.^54^

Grade 3 or worse adverse events (AEs) occurring at a higher incidence (incidence ≥ 2%) in patients receiving chemotherapy plus bevacizumab compared with chemotherapy alone (from data of those phase III trials) included fatigue, hypertension, neutropenia, thrombocytopenia, proteinuria, nausea, headache, dyspnea, epistaxis, abdominal pain, hyponatremia, pain in extremity, and palmar-plantar erythrodysaesthesia syndrome.^55^

### Pazopanib

Pazopanib is an oral TKI of VEGFR-1/-2/-3, PDGF receptor (PDGFR) -α/-β, and c-Kit.^56–58^ Pazopanib showed promising activity in phase I/II trials for patients with platinum-sensitive recurrent OC with increased ORR and PFS.^59–61^ A phase III trial, AGO-OVAR16 (NCT00866697), investigated the efficacy and safety of pazopanib (800 mg daily) as maintenance therapy after first-line chemotherapy in patients with newly diagnosed stage II–IV OC. The study showed that the pazopanib maintenance significantly improved the median PFS (5.6 months longer, *P* = 0.0021).^62^ In subgroup analyses, the PFS benefit with maintenance pazopanib was observed in most subgroups except East Asian patients. To gain further insight, a concurrent study (NCT01227928) similar in design to AGO-OVAR16 was undertaken in the East Asian population, showing that pazopanib maintenance therapy was not associated with a benefit in PFS or OS. There was no satisfactory explanation for this result yet. However, the final analysis of the OVAR16 study was reported in 2019. No difference was observed in the median OS between pazopanib arm and placebo arm.^63^ Grade 3 or worse AEs occurring at a higher incidence in the combined treatment arm compared with placebo included hypertension, neutropenia, diarrhea, thrombocytopenia, increased alanine aminotransferase, and palmar-plantar erythrodysesthesia. A phase I/II trial (NCT02055690) recently reported that combination of pazopanib and fosbretabulin (a prodrug with vascular-disrupting activity) might potentially improve survival outcomes compared with pazopanib alone.^64^ However, this trial was prematurely stopped due to serious cardiac toxicity.

Currently, there are limited data of clinical trials investigating pazopanib for patients with CC or EC. A phase II trial evaluated pazopanib in the treatment of recurrent or persistent carcinosarcoma of the uterus with a result of no response.^65^

### Nintedanib

Nintedanib is another oral TKI of VEGFR-1/-2/-3, FGF receptor (FGFR)-1/-2/–3, and PDGFR-α/β. A phase II trial in platinum-sensitive recurrent OC patients showed an improvement in PFS rate in nintedanib maintenance arm than placebo arm (16.3% vs. 5.0%, *P* = 0.06).^66^ Subsequently, a phase III trial, AGO-OVAR12 (NCT01015118), investigated the combination of nintedanib (200 mg daily) with first-line chemotherapy in patients with newly diagnosed stage IIb–IV OC. The median PFS was 0.6 month longer in the nintedanib arm than that in the placebo arm (*P* = 0.024).^67^ Increased incidences of AEs, including hypertension, gastrointestinal perforation, and bleeding, were reported in the nintedanib arm. The final result of OS is pending. However, for bevacizumab-resistant OC population, single-agent nintedanib was shown to have minimal activity with an ORR of 7.4% in a phase II trial (NCT01669798).^68^

We found limited clinical data of phase II/III trials investigating the activity of nintedanib in EC and CC. One phase II trial, GOG-0229K (NCT01225887), evaluated nintedanib in the treatment of advanced, recurrent, or metastatic EC. It showed modest activity with an ORR of 9.4%.^69^

### Cediranib

Cediranib is a TKI of VEGFR-1/-2/-3 and c-Kit.^70,71^ Given the activity of cediranib in OC showed by early-phase trials,^72^ a phase III trial, ICON6 (NCT00532194), investigated the combination of cediranib (20 mg orally daily) with chemotherapy and as maintenance treatment in patients with platinum-sensitive recurrent OC. The median PFS was 2.3 months longer in the cediranib maintenance arm than that in the placebo arm (*P* < 0·0001).^73^ The data of OS have not been updated. Currently, there are no differences in immature results of median OS across the arms. Increased incidences of diarrhea, neutropenia, hypertension, and voice changes were noted in arms with cediranib.

A phase II study, GOG 229J (NCT01132820), showed cediranib as a monotherapy treatment for recurrent or persistent EC was well-tolerated, with a median PFS of 3.65 months and a median OS of 12.5 months.^74^ Cediranib showed sufficient activity to warrant further investigation for recurrent EC. However, we found limited clinical data for patients with CC.

### Trebananib

Trebananib is a peptide-Fc fusion protein that binds angiopoietin-1/-2, preventing the interaction of angiopoietin with the Tie2 receptor.^75^ Trebananib has shown single-agent activity and prolonged PFS in recurrent OC in early-phase trials.^76,77^ There are three completed phase III trials assessing trebananib in recurrent or newly diagnosed advanced OC. TRINOVA-1 trial (NCT01204749) investigated the addition of trebananib (15 mg/kg intravenously weekly) to single-agent weekly paclitaxel in recurrent OC with platinum-free interval ≤12 months. As a result, the median PFS was 1.8 months longer in the trebananib arm than that in the placebo arm (*P* < 0.0001).^78^ Subsequently, TRINOVA-2 (NCT0128125) evaluated the addition of trebananib to PLD in patients with recurrent OC, and it showed that trebananib did not significantly prolong PFS. However, the addition of trebananib to PLD improved ORR compared with placebo arm (46% vs. 21%, *P* < 0.001).^79^ TRINOVA-3 trial (NCT01493505) showed that the addition of trebananib to fist-line chemotherapy did not improve PFS or produce new safety signals for patients with newly diagnosed advanced OC.^80^ The result of OS was not mature. The major toxic effect associated with trebananib treatment was edema.^78,81^

For recurrent or persistent EC, a phase II trial (NCT01210222) showed an ORR of 3.1%, with insufficient single-agent activity to warrant further investigation of trebananib.^82^

### Other antiangiogenic agents

Apatinib is a small-molecule TKI by binding to the VEGFR-2 ATP-binding site, which is taken orally.^83,84^ Given the promising results of a phase III study in Chinese gastric cancer patients,^85^ apatinib had been actively investigated as a salvage treatment for other advanced solid tumor, including OC.^84^ A phase II study of apatinib in patients with recurrent OC indicated that apatinib (500 mg daily) was a feasible treatment with an ORR of 41.4%.^86^ Grade 3 AEs were hand–foot syndrome, hypertension, and neutropenia. Another phase II trial (NCT02867956) demonstrated that apatinib plus etoposide showed promising efficacy and manageable toxicities in patients with platinum-resistant or -refractory OC with an ORR of 54%.^87^ An ongoing phase III trial in China (NCT04000295) is further evaluating the efficacy and safety of apatinib in patients with platinum-resistant recurrent OC compared with chemotherapy.

Sunitinib and brivanib are oral TKIs of VEGFR and PDGFR. Sunitinib was an FDA-approved drug for renal cell cancer and gastrointestinal stromal tumors. The safety and efficacy of sunitinib in OC were evaluated in several phase II trials with reported ORR ranging from 3.3% to 16.7%.^88–91^ In metastatic or recurrent EC, sunitinib showed promising activity in a phase II trial (NCT00478426) with an ORR of 18.1%.^92^ However, sunitinib had insufficient activity as a single agent in advanced or metastatic CC to warrant further investigation.^93^ Two phase II trials demonstrated that brivanib was well-tolerated and worthy of further investigation in persistent or recurrent EC/CC with an ORR of 7% and 8%, respectively.^94,95^

For the development of antiangiogenic agents and other targeted therapies, the addition of bevacizumab to conventional chemotherapy in OC is a very important step. However, most of the analysis reported so far showed that antiangiogenic agents led to no significant improvement in OS for patients with gynecological cancers. Thus, identification of predictive biomarkers for antiangiogenic agents and development of other targeted drugs are anticipated.

### Poly (ADP-ribose) polymerase (PARP) inhibitors

**Fig. 2:**
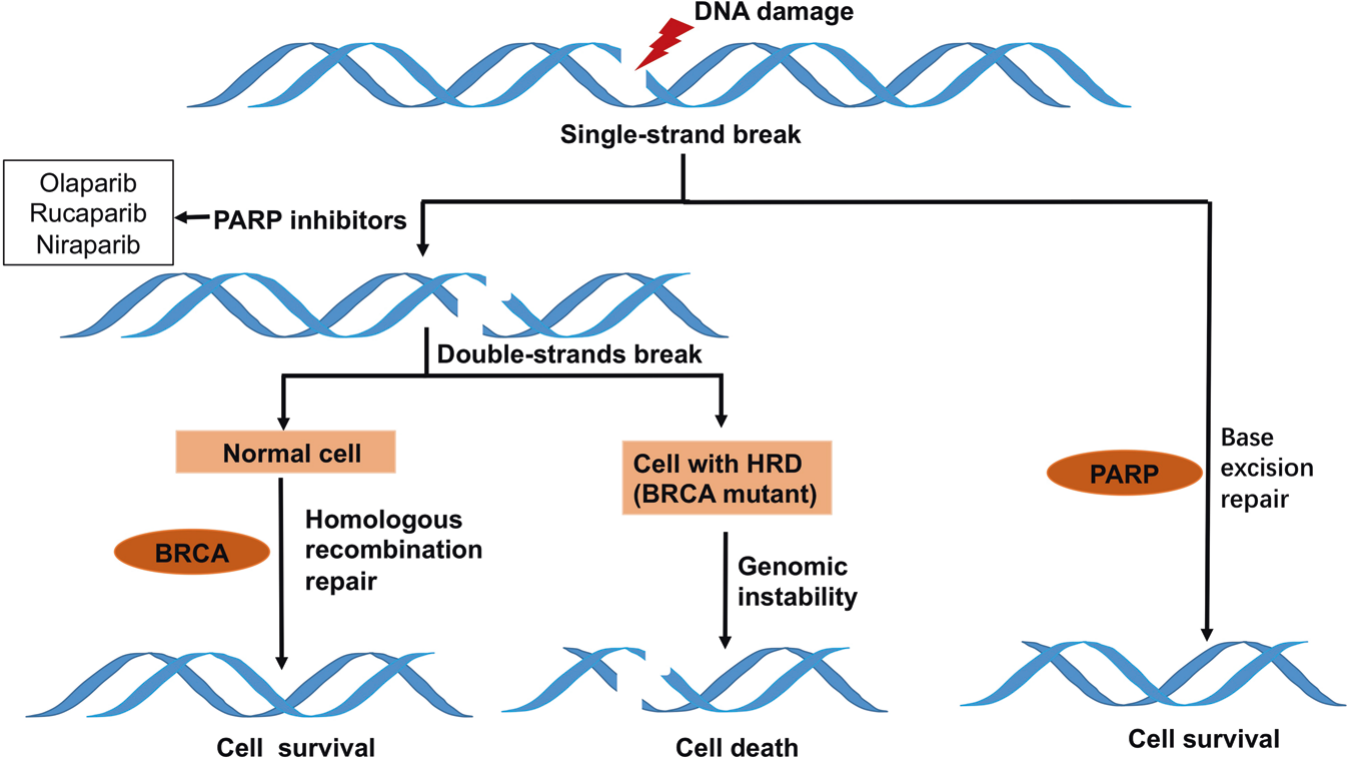
Base-excision repair/single-strand break pathway and the mechanism of synthetic lethal interactions. Inhibition of PARP-1 causes the accumulation of DNA SSBs and ultimately results in DSBs during DNA replication. In cells with HRD, DSBs are left unrepaired or repaired by the error-prone NHEJ pathway, which result in genomic instability and ultimately cell death

PARP is a sort of nuclear enzyme with 17 identified members.^96^ PARP-1 and 2 are involved in DNA repair.^97^ PARP-1, with a structure of the N-terminal zinc-finger DNA-binding domain, the central automodification domain and the C-terminal catalytic domain, was originally found involved in the base-excision repair (BER) pathway, which is important in the repair of single-stranded DNA breaks (SSBs).^98^ Therefore, inhibition of PARP-1 leads to the accumulation of DNA SSBs and ultimately results in DNA double-strand breaks (DSBs) during DNA replication.^99^ DSBs are the most lethal DNA insults. Nonhomologous end joining (NHEJ) and homologous-recombination repair (HRR) are the two main DSB repair pathways in humans.^100^ The preferred pathway is HRR, since it is more accurate. Thus, in cells with functional HRR, PARP inhibition will not result in cell death since DSBs will be precisely and effectively repaired. However, in cells with homologous-recombination deficiency (HRD), such as those with BRCA1/2 mutations, DSBs are left unrepaired or repaired by the error-prone NHEJ pathway, which result in genomic instability and ultimately cell death.^101^ This mechanism of synthetic lethality in HRD cells (Fig. 2) makes PARP inhibitors a novel targeted and personalized cancer treatment.^102,103^

In gynecological cancers, germline and somatic BRCA1/2 mutations (gBRCAm and sBRCAm) occur in ~10–15% of OC patients, and even more frequently in patients with high-grade serous OC (HGSOC), which is the most common type of OC.^22,104,105^ In addition, genomic alterations in other homologous-recombination (HR) genes including ATM, BRIP1, PALB2, and RAD51C are being studied.^106^ The comprehensive genomic analysis has identified that ~50% of high-grade serous tumors (including OC and EC) exhibit HRD.^107,108^ Moreover, the presence of HRD predicts a favorable response to platinum therapies and to PARP inhibitors. PARP inhibitors are also known to sensitize DNA-damaging agents, including carboplatin.^109^ Based on the above facts, PARP inhibitors are supposed to be groundbreaking therapeutic strategies for patients with gynecological cancers, especially for OC.^110^

Several PARP inhibitors, including olaparib, rucaparib, niraparib, veliparib, and talazoparib are actively investigated in clinical trials. The development of PARP inhibitors is productive. Olaparib is the first PARP inhibitor applied in clinic and approved by FDA for cancer treatment, followed by rucaparib and niraparib. The results from phase II/III clinical trials, assessing PARP inhibitors in gynecological cancers, are summarized in Tables 4 and 5. The ongoing clinical trials without results are listed in Table 6.

**Table 4:** Phase III trials (with results) of PARP inhibitors in gynecological cancers

ID	Cancer/condition	No.	Intervention	mPFS (Mos.)	SAEs (%)	Refs
NCT01844986 SOLO-1	OC/BRCAm	319	(1) Placebo	13.8	12.3	^121^
			(2) Olaparib	Not reached, *P* < 0.0001	20.8	
NCT01874353 SOLO-2	OC/recurrent, BRCAm	295	(1) Placebo	5.5	8.08	^120^
			(2) Olaparib	19.1, *P* < 0.0001	17.95	
NCT02477644 PAOLA-1	OC/stage III–IV	806	(1) Bevacizumab+ placebo	16.6	31	^122^
			(2) Bevacizumab+ olaparib	22.1, *P* < 0·0001	31	
NCT01847274 NOVA	OC/platinum-sensitive recurrent	553	(1) Placebo	HRD:10.4; All:8.2	15.08	^138^
			(2) Niraparib	HRD: 21.9; All:13.8, *P < 0.0001	29.97	
NCT02655016 PRIMA	OC/stage III–IV	733	(1) Placebo	8.2	18.9	^140^
			(2) PC + Niraparib	13.8, *P* < 0·0001	70.5	
NCT01968213 ARIEL3	OC/platinum-sensitive recurrent	564	(1) Placebo	BRCAm: 5.4; HRD: 5.4	10.58	^136^
			(2) Rucaparib	BRCAm: 16.6; HRD: 13.6, **P < 0.0001	21	
NCT02470585 GOG-3005	OC/stage III–IV, HGSOC	1140	(1) Placebo	BRCAm: 22.0; HRD: 20.5	32	^150^
			(2) Veliparib combination only	-	27	
			(3) Veliparib throughout	BRCAm: 34.7; HRD: 31.9, ***P < 0.0001	45	

*HRD* homologous-recombination deficiency, *HGSOC* high-grade serous ovarian cancer. *P-value of both HRD cohort and all population are <0.0001. ** and *** *P*-value of both BRCAm and HRD cohorts are <0.0001

**Table 5:** Phase II trials (with results) of PARP inhibitors in gynecological cancers

ID	Cancer/condition	No.	Intervention	ORR (%)	mPFS (mon.)	mOS (mon.)	SAEs (%)	Refs
NCT00494442 STUDY9	OC/advanced, BRCAm	58	Olaparib	33.3	–	–	36.4	^411^
NCT00753545 STUDY19	OC/serous, recurrent	265	(1) Placebo: BRCAm/BRCAwt	4.2	4.3/5.5, *P* < 0.0001	34.9/30.2, *P* = 0.025	8.6	^115,412^
			(2) Olaparib: BRCAm/BRCAwt	12.3	11.2/7.4, *P* = 0.0075	26.6/24.5, *P* = 0.37	22.8	
NCT00679783 STUDY 20	OC/recurrent, HGSOC	91	Olaparib: BRCAm/BRCAwt	41/24	7.4/6.4	–	16	^111^
NCT00628251 STUDY12	OC/advanced, BRCAm	98	(1) Olaparib (200 mg twice daily)	25	5	9	15.6	^413^
			(2) Olaparib (400 mg twice daily)	31.3	5	11	18.8	
			(3) PLD	18.2	4.8	13, All *P* > 0.5	15.6	
NCT01078662 STUDY42	OC/BRCAm	193	Olaparib	31.1	7.03	16.62	30.2	^118^
NCT01081951	OC/advanced or platinum-sensitive recurrent	173	(1) PC	–	9.6	–	20.99	^414^
			(2) Olaparib + PC		12.2, *P* = 0.0012		25.33	
NCT01116648	OC/platinum-sensitive recurrent	90	(1) Olaparib	48.7	8.2	33.3	–	^124,128^
			(2) Cediranib + olaparib	79.6	16.5, *P* = 0.007	44.2, *P* = 0.11	70	
NCT02354586 QUADRA	OC/HGSOC, recurrent, HRD	47	Niraparib	28	5.5	19	56	^141^
NCT02657889 KEYNOTE-162	OC/platinum-resistant recurrent	62	Niraparib + pembrolizumab	18	3.4	Not mature	–	^143^
NCT02354131 ENGOT-ov24	OC/platinum-sensitive recurrent	97	(1) Niraparib	30	5.5	Not mature	–	^142^
			(2) Niraparib + bevacizumab	62	11.9, *P* < 0.0001		65	
NCT01891344 ARIEL2	OC/platinum-sensitive recurrent, HRD	204	Rucaparib: BRCAm	80	12.8	–	24.5	^133^
			BRCAwt, LOH-high	29.3	5.7			
			BRCAwt, LOH-low	10	5.2			
NCT01482715 STUDY10	OC/BRCAm	42	Rucaparib	59.5			76.2	^134^
NCT01306032	OC/HGSOC, BRCAm	75	(1) Cyclophosphamide	19.4	3	–	0	*
			(2) Cyclophosphamide+ veliparib	11.8	3, *P* = 0.68		8.11	
NCT01540565	OC/BRCAm	52	Veliparib	26	8.18	–	20	^146^
NCT01266447	CC/persistent or recurrent	27	Veliparib + topotecan + filgrastim	7	2	8	59.3	^151^

*BRCAwt* BRCA wild-type. *LOH* genomic loss of heterozygosity. *Unpolished data found in ClinicalTrials.gov

**Table 6:** Ongoing phase II–III trials of PARP inhibitors in gynecological cancers (not including novel combination therapy)

ID	Cancer/condition	Setting	No.	Start date	Intervention	Phase/assignment	Status
NCT02282020 SOLO-3	OC/platinum-sensitive recurrent, BRCAm	Maintenance	266	2015.2	Olaparib vs. single-agent chemotherapy	III/randomized, parallel	Active, not recruiting
NCT03402841 OPINION	OC/platinum-sensitive recurrent, without BRCAm	Maintenance	279	2018.1	Olaparib	III/single group	Active, not recruiting
NCT03534453 L-MOCA	OC/platinum-sensitive recurrent	Maintenance	300	2018.5	Olaparib	III/single group	Active, not recruiting
NCT02855944 ARIEL4	OC/recurrent	Monotherapy	345	2016.9	Rucaparib vs. chemotherapy	III/randomized, crossover	Recruiting
NCT04227522 MAMOC	OC/advanced	Maintenance	190	2020.1	Rucaparib vs. placebo	III/randomized, parallel	Not yet recruiting
NCT03519230	OC/platinum-sensitive recurrent	Maintenance	216	2018.5	Pamiparib vs. placebo	III/randomized, parallel	Recruiting
NCT03709316	OC/advanced	Maintenance	381	2018.6	Nirapairb vs. placebo	III/randomized, parallel	Recruiting
NCT03863860	OC/platinum-sensitive recurrent	Maintenance	216	2019.1	Fluzoparib vs. placebo	III/randomized, parallel	Not yet recruiting
NCT04169997	OC/advanced	Maintenance	393	2020.2	IMP4297 vs. placebo	III/randomized, parallel	Recruiting
NCT02489006	OC/recurrent	Neoadjuvant	24	2016.7	Olaparib vs. platinum-based chemotherapy	II/ randomized, parallel	Recruiting
NCT03470805	OC/recurrent, after PLD	Maintenance	9	2018.6	Olaparib	II/ single group	Active, not recruiting
NCT04377087	OC/recurrent	Delayed maintenance	75	2020.5	Olaparib	II/ single group	Not recruiting
NCT03016338	EC/recurrent	–	44	2017.11	Niraparib	II/ single group	Recruiting
NCT03644342	CC/metastatic invasive	Concurrently	20	2019.7	Niraparib + radiotherapy	II/ single group	Recruiting
NCT03891576	OC/platinum-sensitive recurrent	Maintenance	105	2019.10	Niraparib	II/ single group	Not yet recruiting
NCT04217798	OC/platinum-resistant or -refractory	Maintenance	32	2020.1	Niraparib + etoposide	II/ single group	Not yet recruiting
NCT03617679	EC/metastatic and recurrent	Maintenance	138	2019.3	Rucaparib vs. placebo	II/randomized, parallel	Recruiting
NCT03795272	CC/locally advanced	Maintenance	162	2019.11	Rucaparib vs. placebo	II/randomized, parallel	Withdrawn
NCT04171700 LODESTAR	Solid tumor/HRD	–	220	2019.11	Rucaparib	II/ single group	Recruiting
NCT03509636	OC/recurrent, BRCAm	–	113	2018.4	Fluzoparib	II/ single group	Active, not recruiting

### Olaparib

Olaparib is the best studied PARP inhibitor and approved by FDA for the maintenance treatment of selected advanced or recurrent OC patients. Early-phase clinical trials of olaparib demonstrated activity signals in patients with OC, with favorable tolerance and response rates.^58,111–113^ Following these promising results,^114^ a notable randomized placebo-controlled phase II trial, Study 19 (NCT00753545), evaluated olaparib as maintenance monotherapy for patients with platinum-sensitive recurrent OC. The median PFS was significantly longer in the olaparib arm compared with placebo (3.6 months longer, *P* < 0.001).^115^ A retrospective preplanned analysis suggested that patients with BRCAm gained the greatest PFS benefits from olaparib treatment (6.9 months longer, *P* < 0.0001). An exploratory post hoc analysis of Study 19 also suggested a numerical improvement in the OS.^116^ Although the PFS benefit was less in patients without BRCAm (1.9 months longer, *P* = 0.0075), this significant benefit suggested that a proportion of patients without BRCAm might also benefit from olaparib treatment.^117^ Another single-arm phase II trial, Study 42 (NCT01078662), evaluated olaparib as treatment for cancer patients with gBRCAm, including ovarian, breast, prostate, and pancreatic cancer. The ORR was 31.1% in platinum-resistant recurrent OC cohort. Stable disease (SD) was seen in 40% of patients, confirming significant activity.^118,119^ Based on these findings, the FDA approved single-agent olaparib as recurrence therapy for patients with advanced OC with gBRCAm who have received three or more lines of chemotherapy in 2014.

Several large randomized phase III trials of olaparib in gynecological cancers (mainly in OC) are currently in progress. The following three of the phase III trials reported promising results in OC. SOLO-2 trial (NCT01874353) evaluated the efficacy of olaparib as maintenance therapy in platinum-sensitive recurrent OC patients with BRCAm who had received at least two lines of previous chemotherapy. The results demonstrated a statistically significant improvement in investigator-assessed median PFS in the olaparib arm compared with placebo (13.6 months longer, *P* < 0·0001). At the time of the analysis of PFS, OS data were not mature with 24% of events.^120^ Based on this trial, the FDA approved olaparib as maintenance therapy for women with recurrent OC who are in complete or partial response to platinum-based chemotherapy in 2017. Another phase III trial, SOLO-1 (NCT01844986), evaluated the efficacy of olaparib as maintenance therapy in newly diagnosed advanced OC patients with BRCAm.^121^ After a median follow-up of 41 months, the risk of disease progression or death was 70% lower with olaparib than with placebo (*P* < 0.001). The estimated median PFS was not reached in the olaparib arm versus 13.8 months in the placebo arm (*P* < 0.0001). At the time of the analysis, OS data were not mature. Following this study, the FDA approved olaparib as maintenance therapy of advanced OC patients with BRCAm, who are in complete or partial response to first-line platinum-based chemotherapy in 2018. At the ESMO Congress 2019, new findings of a phase III trial, PAOLA-1/ENGOT-ov25 (NCT02477644), were presented. This is the first phase III trial to evaluate efficacy and safety of a PARP inhibitor plus bevacizumab as first-line maintenance therapy in advanced OC not restricted by surgical outcome or BRCA status. According to the results, patients with newly diagnosed OC had significantly improved the median PFS with addition of olaparib to bevacizumab maintenance treatment, as compared to placebo plus bevacizumab following first-line chemotherapy (5.5 months longer, *P* < 0.0001).^122^ Moreover, the PFS benefit in subgroups of patients with BRCAm and patients with other HRD was even more obvious (19.5 months longer and 11.5 months longer, respectively). In PAOLA-1 trial, the rate of AEs leading to treatment discontinuation is the highest figure reported across PARP inhibitor trials. However, there was no impact in QOL.

The FDA-recommended olaparib dose is 300 mg (two 150 mg tablets) taken orally twice daily. The most common serious AEs reported in SOLO-1 and SOLO-2 were anemia and neutropenia.

There are other three ongoing phase III trials of olaparib (as monotherapy) registered in the ClinicalTrials.gov database without available results, including SOLO-3 (NCT02282020), OPINION (NCT03402841), and L-MOCA (NCT03534453) (Table 6).

A phase II trial (NCT01116648) evaluated the efficacy and toxicity of the combination of cediranib and olaparib compared to olaparib alone in platinum-sensitive recurrent OC, based on the data from early clinical trial.^123–126^ This novel combination of angiogenesis inhibitor and PARP inhibitor improved the median PFS by 8.3 months compared with PARP inhibitor alone (*P* = 0.007).^124,127^ In the updated analysis in 2019, subset analyses within stratum defined by BRCA status demonstrated that this combination therapy significantly improved both median PFS (23.7 vs. 5.7 months, *P* = 0.002) and median OS (37.8 vs. 23.0 months, *P* = 0.047) in gBRCAwt/unknown patients.^128^ It encouraged the novel combination therapy of different targeted agents explored as a potential treatment strategy. Currently, we found only clinical case reports about efficacy of olaparib in other gynecological cancers (e.g., EC).^129^

### Rucaparib

Rucaparib is a potent, oral, small-molecule PARP inhibitor.^130,131^ Rucaparib was FDA-approved in 2016 as monotherapy for the treatment of recurrent OC patients with BRCAm who have been treated with two or more chemotherapies. This approval was grounded on the proportion of patients with a favorable ORR observed in a pooled population of patients with BRCAm high-grade OC from the Study 10 and ARIEL2 trials.^132–135^ ARIEL2 (NCT01891344) is a phase II trial, assessing rucaparib as recurrence therapy for patients with platinum-sensitive OC. The median PFS after rucaparib treatment was 7.6 months longer in the BRCAm subgroup (*P* < 0.0001).

In a phase III trial, ARIEL3 (NCT01968213), assessed the efficacy and safety of rucaparib as maintenance therapy in patients with platinum-sensitive recurrent OC. The median PFS in patients with BRCAm was 11.2 months longer in the rucaparib arm than that in the placebo arm (*P* < 0·0001). In patients with HRD, it was 8.2 months longer (*P* < 0·0001). In the intention-to-treat (ITT) population, the median PFS was 5·4 months longer in patients in the rucaparib arm than that in the placebo arm (*P* < 0·0001).^136^ Based on this study, the FDA approved rucaparib for the maintenance treatment of recurrent OC patients who are in a complete or partial response to platinum-based chemotherapy. The ongoing ARIEL4 trial (NCT02855944) is another phase III study of rucaparib compared with chemotherapy in recurrent OC patients with BRCAm after two or more prior lines of therapy. The combination of rucaparib with other novel therapies (e.g., immune checkpoint inhibitor) is investigated for OC and EC in Phase I/II trials (NCT03101280, NCT03572478). A new phase III trial, MAMOC (NCT04227522), is going to investigate rucaparib maintenance therapy after bevacizumab maintenance following first-line chemotherapy in advanced OC.

The FDA-recommended rucaparib dose is 600 mg (two 300 mg tablets) taken orally twice daily. The most common serious AEs reported in ARIEL3 were anemia, pyrexia, vomiting, and small intestinal obstruction.

### Niraparib

Niraparib is another FDA-approved PARP inhibitor.^137^ A phase III trial, ENGOT-OV16/NOVA (NCT01847274), evaluated the efficacy of niraparib as maintenance treatment for patients with platinum-sensitive recurrent OC. The results showed that niraparib increased PFS regardless of BRCA status when compared with placebo. Patients in the niraparib arm had significantly longer median PFS than those in the placebo arm, including 21.0 vs. 5.5 months in the gBRCAm cohort, 12.9 months vs. 3.8 months in the non-gBRCAm cohort for patients who had tumors with HRD, and 9.3 months vs. 3.9 months in the overall non-gBRCAm cohort (*P* < 0.001 for all three comparisons).^138^ Based on this study, niraparib was approved by FDA in 2017 as maintenance therapy for adult patients with recurrent OC who are in complete or partial response to platinum-based chemotherapy.^138^ Furthermore, A retrospective subanalysis demonstrated the safety and efficacy of niraparib in the subgroup of patients aged ≥70 years in this trial, suggesting that the use of niraparib should be considered in this population.^139^ Findings from another phase III trial, PRIMA (NCT02655016), were presented at the ESMO Congress 2019, and recently reported. This study evaluated the efficacy of niraparib following first-line chemotherapy in patients with newly diagnosed advanced OC and had similar findings with NOVA trial. Patients in the niraparib arm had substantial improvement in the median PFS compared to those in placebo arm (5.6 months longer, *P* < 0.0001). In the HRD cohort, the improvement of the median PFS was even greater in treatment group (21.9 vs. 10.4 months, *P* < 0.0001).^140^ Another phase III trial (NCT03709316) of niraparib in advanced OC is under way (Table 6). Several other phase II trials are studying the potential role of niraparib in different clinical settings. QUADRA trial (NCT02354586) assessed the activity of single-agent niraparib as the fourth or later line treatment for patients with platinum-sensitive recurrent HGSOC.^141^ This study met the primary endpoint, with an ORR of 28% in HRD-positive population. The median PFS in this population was 5.5 months. The median OS was 26 months in the BRCAm population, 19.0 months in the HRD-positive population, and 15.5 months in the HRD-negative population. NSGO-AVANOVA2/ENGOT-OV24 trial (NCT02354131) showed that niraparib (300 mg orally daily) plus bevacizumab (15 mg/kg intravenously every 3 weeks) significantly improved the median PFS compared with niraparib alone in patients with platinum-sensitive recurrent OC (5.4 months longer, *P* < 0.00001).^142^ TOPACIO/KEYNOTE-162 trial (NCT02657889) evaluated niraparib (200 mg orally daily) combined with pembrolizumab (an immune checkpoint inhibitor, 200mg intravenously on day 1 of each 21-day cycle) in patients with recurrent OC. The ORR was 18%, with a disease control rate of 65%. This novel combination therapy was tolerable, and responses in patients without HRD were higher than expected with either agent as monotherapy.^143^

The FDA-recommended niraparib dose is 300 mg taken orally once daily. The most common serious AEs reported in NOVA and PRIMA were thrombocytopenia, anemia, and neutropenia. Disutility analyses showed no significant QOL impairment associated with these toxic effects.^144^

### Veliparib

Veliparib is a potent small-molecule inhibitor of PARP-1/2.^145^ Early-phase trials demonstrated activity of veliparib among OC patients with BRCAm to provide rationale for further clinical development.^109,146–149^ New results from a phase III trial, VELIA/GOG-3005 (NCT02470585), were reported at the ESMO Congress 2019. It assessed the efficacy of veliparib (150 mg orally twice daily) added to first-line chemotherapy and continued as maintenance monotherapy in patients with previously untreated advanced HGSOC. In the BRCAm cohort, the median PFS was 12.7 months longer in the veliparib-throughout arm than in the control arm (*P* < 0.001). In the HRD cohort, it was 11.4 months longer (P < 0.001). And in the ITT population, the median PFS was 5.2 months longer (*P* < 0.001). AEs reported with veliparib were predominantly gastrointestinal and hematologic. The most common AE leading to the discontinuation of veliparib was nausea.^150^

For the treatment of CC, there was a phase I/II trial (NCT01266447) that assessed veliparib in combination with topotecan for patients with recurrent or persistent CC, showing minimal clinical activity with an ORR of 7%.^151^ Another phase I trial (NCT01281852) investigated veliparib in combination with cisplatin and paclitaxel in patients with recurrent or metastatic CC.^152^ The results demonstrated an ORR of 34%, illustrating the potential of PARP inhibitors as a combination therapy in CC.

### Other PARP inhibitors

Talazoparib is a potent PARP inhibitor showing antitumor cytotoxicity at much lower concentrations than other agents, with an ORR of 42% in early-phase clinical trials for advanced OC with BRCAm.^153,154^

Pamiparib is a highly selective oral PARP-1/2 inhibitor capable of penetrating the brain.^155^ In a phase I trial of pamiparib combined with tislelizumab (an immune checkpoint inhibitor) in advanced solid tumors, 9 (26%) of the 34 patients with OC achieved clinical responses.^156^ A phase II trial (NCT03933761) is assessing the clinical benefit rate of pamiparib in fusion-positive, reversion-negative HGSOC with BRCAm.

Fluzoparib is a novel PARP inhibitor undergoing clinical trials with potent anticancer activities.^157,158^ Two ongoing phase III trials (NCT03519230 and NCT03863860) are investigating the efficacy of pamiparib and fluzoparib as maintenance therapy in recurrent OC, respectively.

In summary, PARP inhibitors are acting as an exciting new option for patients with OC by significantly increasing both PFS and OS, especially for those with HRDs. However, cost effectiveness and drug resistance remain to be improved.^159,160^ In the future, it is necessary to identify more indications and predictive biomarkers.^161,162^ Moreover, numerous ongoing clinical trials of novel combination therapies are guiding the future direction of targeted therapy strategies (Tables 13 and 14).^163,164^

### PI3K/AKT/mTOR pathway blockade

The phosphatidylinositol 3-kinase/protein kinase B/mammalian target of rapamycin (PI3K/AKT/mTOR) signaling is one of the critical intracellular pathways that regulates important cell activities, such as cell growth, survival, proliferation, differentiation, metabolism, apoptosis, and angiogenesis.^165^ PI3K is plasma membrane-associated lipid kinases, composed of regulatory subunit (PIK3R) and catalytic subunit (PIK3CA) that mediate receptor binding, activation, and localization of the enzyme.^166^ In normal conditions, PI3K can be activated by a variety of stimuli, including growth factors, cytokines, and hormones.^167^ Activation of AKT regulates a number of downstream targets. mTOR is a serine/threonine protein kinase and the best-described downstream target of AKT, composed of mTOR Complex 1 (mTORC1) and mTOR Complex 2 (mTORC2).^168^ mTORC1 is sensitive to inhibition by rapamycin, and its analogs and mTORC2 exerts a positive feedback activation on AKT.^169^ There are also endogenous negative regulators of the PI3K pathway, such as the tumor suppressor—phosphatase and tensin homologue (PTEN).^170^ The PI3K/Akt/mTOR pathway is also involved in cross talk with other signaling pathways, including the Ras/Raf/MEK and estrogen receptor (ER) pathways.^171^ The overview of the PI3K/AKT/mTOR signaling pathway is included in Fig. 1. In cancer, this pathway can be aberrantly activated via a number of mechanisms, including loss of tumor-suppressor function, exposure to carcinogens, mutations/amplifications of PI3K, and mutations/amplifications of AKT. The deregulation of the PI3K/ AKT/mTOR pathway occurs in many cancers.^172–174^ As for gynecological cancers, this pathway is overactivated in OC (~70%),^175–177^ as well as EC and CC.^178–180^ In EC, the mutation rates of PI3K and PTEN were high, especially in the POLE subgroup.^20^ In vitro model of CC, mTOR inhibitors markedly reduced the expression level of HPV E7 protein, inducing apoptosis.^181^ Based on the preclinical evidence, the PI3K/AKT/mTOR pathway emerges as a potential therapeutic target in cancer, as well as gynecological malignancy.^176,182,183^ There are many drugs being tested in each part of this pathway: PI3K inhibitors, mTOR inhibitors, AKT inhibitors, and dual inhibitors on PI3K/mTOR or PI3K/AKT. mTOR inhibitors (everolimus and temsirolimus) and PI3K inhibitors (idelalisib, alpelisib and copanlisib) have been FDA-approved to be effective in the advanced cancer treatment, such as breast cancer, renal cell carcinoma, and lymphoma.^164^ Despite there are a number of preclinical/clinical data on PI3K/AKT/mTOR pathway inhibitors, currently there is no FDA-approved indication in gynecological cancers.

### mTOR inhibitors

The most tested drugs in the PI3K/AKT/mTOR pathway are those blocking mTOR activity. Temsirolimus, everolimus, and ridaforolimus are the most-studied mTOR inhibitors in gynecological cancers. The results of completed clinical trials (phase II) investigating the safety and efficacy of them in gynecological cancers are summarized in Table 7.

**Table 7:** Completed phase II trials of PI3K/AKT/mTOR pathway inhibitors in gynecological cancers

ID	Cancer/condition	No.	Intervention	ORR (%)	CBR (%)	mPFS (mon.)	mOS (mon.)	SAEs (%)	Refs
NCT001460979	EC/advanced	22	Temsirolimus	10	35	3.0	21.3	–	^415^
AGO-GYN8	OC/advanced	22		4.8	38.1	3.4	21.9		
NCT00429793	OC/recurrent	54	Temsirolimus	9.3	–	3.1	11.6	9.26	^416^
NCIC IND 160	EC/recurrent or metastatic	23	Temsirolimus	26	89	–	–	–	^188^
NCT00723255	EC/recurrent	53	Temsirolimus + bevacizumab	24.5	40	5.6	16.9	63.27	^417^
NCT00729686	EC/advanced or recurrent	71	(1) Temsirolimus	22	52.4	4.9	10.8	36	^187^
			(2) Temsirolimus + hormone therapy	14.3	–			61.9	
NCT00072176 NCIC CTG	EC/locally advanced, recurrent, or metastatic	60	(1) Temsirolimus + hormone therapy	14	89	7.33	–	33.33	^418^
			2) Temsirolimus + chemotherapy	4	50	3.25		33.33	
NCT00977574 GOG-86P	EC/stage III–IV or recurrent	349	(1) Bevacizumab + PC	59.5	–	–	34	42.8	^197^
			(2) Temsirolimus + PC	55.3			25	50.4	
			(3) Bevacizumab + IC	52.9			25.2	46.5	
NCT01026792 NCIC IND199	CC/advanced or metastatic	38	Temsirolimus	3	60.6	3.52	–	40.5	^419^
NCT00087685	EC/progressive or recurrent	35	Everolimus	21	45.1	–	–	–	^192^
NCT01068249	EC/recurrent	38	Everolimus + letrozole	32	40	3	14	31.6	^194^
NCT01797523	EC/recurrent	58	Everolimus + letrozole + metformin	29	66.7	–	–	–	^193^
NCT02283658	OC/ER + , recurrent	20	Everolimus + letrozole	16	37	3.9	13	63	^420^
NCT00739830	EC/stage III–IV	130	(1) Hormone or chemotherapy	4	17	1.9	–	34	^421^
			(2) Ridaforolimus	0	35	3.6		57	
NCT00122343	EC/recurrent	45	Ridaforolimus	11	19	–	–	33	^422^
NCT00770185	EC/recurrent	35	Ridaforolimus	8.8	62	–	–	37.1	^423^
–	EC/progressive	45	Ridaforolimus	7.4	33	–	–	35.6	^424^
NCT01935973	EC/recurrent or persistent	26	GSK2141795 + trametinib	8.3	–	–	–	61	^203^
NCT02538627	CC/persistent or recurrent	35	GSK2141795 + trametinib	7.1	44	3.6	14.8	57	^202^
NCT01307631	EC/recurrent	37	MK2206	5.5	33	–	8	37.84	^205^
NCT01397877 ENDOPIK	EC/advanced or recurrent	40	BKM120	0	60	4.5		21	^209^
NCT02193633	OC/HGSOC	27	Vistusertib + chemotherapy	52	78	5.8	–	–	^198^
NCT01587040	EC/advanced or recurrent	67	Pilaralisib	6	13.4	–	–	52.9	^210^
NCT01420081	EC/recurrent	40	Gedatolisib	16	5	3.6	–		^212^

*CBR* clinical benefit rate = complete response + partial response + stable disease, *ER+* estrogen receptor positive

Consistent with preclinical findings,^171,184–186^ initial clinical trials demonstrated promising activities of mTOR inhibitors in EC. Temsirolimus, an intravenous mTORC1 inhibitor (25 mg weekly), showed efficacy as monotherapy for advanced and recurrent EC with ORRs of 22–25%.^187–189^ Ridaforolimus is another intravenous mTORC1 inhibitor, administrated at a dose of 12.5 mg daily for 5 consecutive days every 2 weeks, showing a modest therapeutic efficacy as a single agent.^190^ A phase II trial studied the efficacy and tolerability of ridaforolimus in recurrent and advanced EC with an ORR of 8.8% and a SD of 52.9%.^191^ Everolimus, an oral mTORC1 inhibitor (10 mg daily), was evaluated in a phase II study (NCT00087685) for the treatment of patients with recurrent or persistent EC, showing an ORR of 0% and a SD of 43%.^192^ However, everolimus was reported to have the best effects in recurrent EC when combined with hormonal therapy (e.g., letrozole, an aromatase inhibitor), showing ORRs of 29–32%.^193,194^ Given that mTOR inhibitors are cytostatic cell cycle agents with a benefit mainly in terms of disease stabilization rather than disease response (tumor shrinkage), we found only modest effects of mTOR inhibitors as monotherapy in OC and CC based on current clinical evidence.^195^ Reasons to these disappointing results might be: (1) one pathway blockade is insufficient; combined therapies are needed; (2) analogs of rapamycin selectively inhibit mTORC1; the other mTOR complex, mTORC2, is a positive regulator of AKT; (3) predictive biomarkers are required to identify population who can get most benefit from this pathway blockade. Considering the evidence from preclinical studies showing promising activity of mTOR inhibitors in combination with chemotherapy, a number of clinical trials assessed the efficacy of the addition of mTOR to cytotoxic drugs, as well as novel combination of different targeted therapies. A Phase II trial (NCT01031381), evaluating everolimus plus bevacizumab in recurrent OC, reported that 28% patients were progression-free at 6 months. Patients with both platinum-sensitive and -resistant disease showed response. Overall, the regimen was well-tolerated.^196^ A randomized Phase II trial (NCT00977574) compared the efficacy of temsirolimus in combination with chemotherapy (carboplatin and paclitaxel) to bevacizumab plus chemotherapy in advanced or recurrent EC. Patients treated by temsirolimus plus chemotherapy had an ORR of 55.3%, and a median OS of 25 months. However, the results reported no improvement in comparison to bevacizumab plus chemotherapy.^197^ A phase I trial (NCT02193633) investigated the efficacy of vistusertib (a dual mTORC1/mTORC2 inhibitor) in combination with paclitaxel in OC, showing an ORR of 52% and a median PFS of 5.8 months.^198^ Currently, no specific predictive biomarker has been recognized. Tumors with PI3K or PTEN mutations did not necessarily respond to mTOR inhibitors.^199^ Common treatment-related AEs of mTOR inhibitors include stomatitis, mucositis, pneumonitis, rash, fatigue, anemia, diarrhea, nausea, vomiting, hyperglycemia, and immunosuppression.

### AKT inhibitors

GSK2141795 and MK2206 are inhibitors targeting AKT, acting upstream of mTOR.^200,201^ A phase II trial tested dual inhibition of PI3K and Ras signaling by combining the AKT inhibitor (GSK2141795, 50 mg orally daily) and the MEK inhibitor (trametinib, 1.5 mg orally daily) in recurrent CC, with AEs including gastrointestinal events, fatigue, and rash. One patient had an unconfirmed partial response, with an ORR of 7.1%. Eight patients (57.1%) had stable disease.^202^ However, the combination of trametinib and GSK2141795 was shown to have high levels of toxicity in EC at this dose. And the preliminary efficacy is disappointing in another phase II trial (NCT01935973).^203,204^ Moreover, a two-arm, PIK3CA mutation stratified phase II trial (NCT01307631) in recurrent EC demonstrated limited single-agent activity of MK2206 (200 mg orally weekly) in both PIK3CA mutant and wild-type populations.^205^ Afuresertib, another AKT inhibitor, combined with chemotherapy showed an acceptable safety profile in patients with platinum-resistance OC in a phase I study.^206^ A phase II trial of afuresertib plus weekly paclitaxel in platinum-resistance OC (NCT04374630, PROFECTA-II) is under way.

### PI3K inhibitors

BKM120 (buparlisib) is an oral pure PI3K inhibitor. It was shown to have antitumor activity in preclinical and early trials.^207,208^ However, a phase II trial (NCT01397877) demonstrated that the BKM120 (100 mg orally daily) was associated with a minimal antitumor activity as monotherapy in advanced or recurrent EC.^209^ Another oral PI3K inhibitor, pilaralisib (600-mg capsules or 400-mg tablets daily), also had minimal success in a phase II trial in advanced or recurrent EC.^210^ PF-04691502 and gedatolisib (PF-05212384) are potent, dual PI3K/mTOR inhibitors.^211^ A randomized phase II non-comparative trial (NCT01420081) was conducted in patients with recurrent EC following platinum-containing chemotherapy. Clinical benefit response criteria were only met in the gedatolisib/stathmin-low arm.^212^ Common treatment-related AEs include nausea, mucositis, decreased appetite, diarrhea, fatigue, vomiting, rash, and stomatitis.

In summary, the role of the PI3K/AKT/mTOR pathway inhibitors in gynecological cancers is not yet clear. The reasons for the unsatisfactory results may be related to the feedback loops and compensatory activation of Ras pathway. Even though the presented clinical results are controversial,^213^ there are amount of preclinical studies and clinical trials in progress, mainly combining PI3K signaling blockade with other therapies or different targeted agents.^214^ For example, a randomized phase II trial (NCT02397083) is designed to study how everolimus works with the levonorgestrel-releasing intrauterine system for early-stage EC. Another phase II trial (NCT03008408) is to learn if the combination of everolimus, letrozole, and ribociclib (a CDK4/6 inhibitor) can help to control recurrent or progressive EC. Dual mTORC inhibition continues to be assessed in advanced or recurrent OC (NCT03648489). Furthermore, for the future of this pathway targeted therapy, studies of predictive biomarkers might be very helpful and important.

### Human epidermal growth factor receptor-targeted inhibitors

**Fig. 3:**
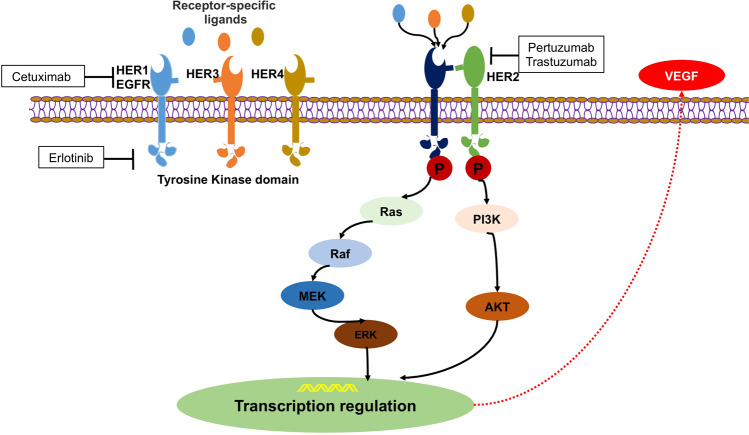
The HER signal transduction pathway and therapeutic interventions

Human epidermal growth factor receptors (HERs), also known as erythroblastic leukemia viral oncogene (erbB) family, include HER1(Erb1, EGFR), HER2 (Erb2), HER3 (Erb3), and HER4 (Erb4).^215^ Structural features of HER proteins include extracellular ligand-binding domain, transmembrane domain, and intracellular protein tyrosine kinase domain.^216^ When ligands bind to the extracellular domain, HERs form homodimers or heterodimers with other members of the family.^217^ As an exception, HER2 does not bind any ligand, but it has the most favorable kinase activity. HER3 lacks tyrosine kinase activity.^218^ Dimerization of ligand-activated HERs initiates a cascade of downstream signaling, such as PI3K/AKT/mTOR, Ras/Raf/MAPK (the mitogen-activated protein kinase pathway), and JAK/ STAT (the signal transducer and activation of the transcription pathway), which regulate from cell division to death, motility to adhesion (Fig. 3).^216^ Overexpression of EGFR and HER2 protein and amplification of HER2 oncogene play an important role in carcinogenesis, associated with breast, lung, gastric, ovarian, endometrial, and bladder cancer.^219–222^ HER2 is also related to increased recurrence and poor prognosis in some cancers.^223^ Thus, EGFR and HER2 are promising targets for treatment of cancer.^224–227^

HER-targeted drugs include monoclonal antibodies and small-molecule inhibitors. Monoclonal antibodies against the extracellular domain of the HER receptor include cetuximab, nimotuzumab, trastuzumab, pertuzumab, and ado-trastuzumab emtansine (T-DM1).^227–229^ Cetuximab and nimotuzumab bind to the extracellular domain of the EGFR. Trastuzumab obstructs HER2 homodimerization. HER2 overexpression is required for trastuzumab to be effective.^230^ Pertuzumab inhibits HER2 heterodimerization and does not require HER2 overexpression to be effective.^231^ T-DM1 is trastuzumab conjugated to emtansine (a microtubule inhibitor), which inhibits microtubule assembly in the cytoplasm and thus leads to cell death.^232^ Small-molecule inhibitors are TKIs including gefitinib, erlotinib, lapatinib, and afatinib against intracellular kinase domain to prevent signaling.^233^ Among them, gefitinib and erlotinib are inhibitors selective for EGFR.^234,235^ Lapatinib and afatinib inhibit both EGFR and HER2.^236^ Most of them have been approved by FDA as targeted therapies for certain advanced or recurrent cancers with selected biomarkers, such as breast cancer, colorectal cancer and non-small cell lung cancer (NSCLC).

As for gynecological cancers, HER2 is an important oncogene in high grade and stage EC, especially in uterine serous carcinoma.^237,238^ In OC, the rate of HER2 overexpression is highly variable (ranging from 2% to 66%), and the rate of EGFR overexpression is 30–70%.^239,240^ In CC, the rate of EGFR overexpression ranges from 6% to 90%.^241,242^ However, unlike in NSCLC,^243^ the clinical significance of EGFR/HER2 gene amplification or protein overexpression and the efficacy of HER-targeted therapy are still controversial in gynecological cancers (Table 8).

**Table 8:** Completed phase II–III trials of HER-targeted therapy in gynecological cancers

ID	Cancer/condition	Phase	No.	Intervention	ORR (%)	mPFS (mon.)	mOS (mon.)	SAEs (%)	Refs
NCT02095119	CC/recurrent or metastatic	I/II	17	Nimotuzumab	0	5.43	9.9	–	^425^
NCT00997009 MITO CERV-2	CC/recurrent	II	108	(1) PC	84.6	5.2	17.7	–	^244^
				(2) PC + cetuximab	76.4	7.6, *P* = 0.20	17, *P* = 0.27		
NCT10101192	CC/advanced, persistent, or recurrent	II	27	Cetuximab + cisplatin	29.6	3.91	8.77	–	^245^
NCT00499031	CC/persistent or recurrent	II	38	Cetuximab	0	1.97	6.7	42.86	^426^
NCT00086892	OC/platinum-sensitive recurrent	II	29	Cetuximab	32.1	9.4	–	–	^248^
NCT01684878	OC/platinum-resistant, with low tumor	III	156	(1) Placebo + chemotherapy	8.7	2.6	8.4	37.66	^253,254^
PENELOPE	HER3 mRNA expression			(2) Pertuzumab + chemotherapy	13.1	4.3, *P* = 0.14	10.2, *P* = 0.60	43.42	
NCT02004093	OC/recurrent	II	149	(1) Chemotherapy	-	9.3	Not reached	16.2	*
				(2) Pertuzumab + chemotherapy		8.0, *P* = 0.3967	28.2	26.7	
NCT00096993	OC/platinum-resistant recurrent	II	103	(1) Placebo + chemotherapy	4.6	2.6	13.1	61.54	^252^
				(2) Pertuzumab + chemotherapy	13.8	2.9, *P* = 0.07	13.0, *P* = 0.65	35.38	
NCT02004093	OC/platinum-sensitive recurrent	II	149	(1) PC	–	9.3	Not yet estimable	16.22	^255^
				(2) PC + pertuzumab	–	8.5	28.2	26.67	
NCT00189579	OC/recurrent or refractory, HER2 +	II	41	Trastuzumab	7.3	2.0	–	–	^239^
NCT00006089	EC/recurrent or stage III–IV, HER2 +	II	34	Trastuzumab	0	1.8	6.8	–	^249^
NCT01367002	EC/advanced or recurrent, serous	II	61	(1) Chemotherapy	75	8.0	–	51	^250^
				(2) Trastuzumab + chemotherapy	44	12.6, *P* = 0.005			
NCT00023699	OC/persistent or recurrent	II	30	Gefitinib	0	1.23	3.7	–	^427^
NCT00189358	OC/platinum-resistant recurrent	II	56	Gefitinib + tamoxifen	0	1.9	8.4	3.6	^428^
-	CC/advanced or metastatic	II	28	Gefitinib	0	1.2	3.6	–	^266^
NCT00113373	OC/recurrent	II	28	Lapatinib	0	8.0	–	40	^258^
NCT00436644	OC/platinum-resistant recurrent	II	18	Lapatinib + topotecan	5.6	3.5	15.5	22.2	^260^
NCT00888810	OC/recurrent	II	39	Lapatinib + topotecan	14	–	–	–	^259^
NCT00096447	EC/persistent or recurrent	II	30	Lapatinib	3.3	1.82	7.33	33.3	^256^
NCT00430781	CC/metastatic	II	230	(1) Lapatinib	5	4.0	9.1	28.95	^257^
				(2) Pazopanib	9	4.2, *P* < 0.013	11.8, *P* = 0.045	37.84	
NCT00263822	OC/no progression after first-line PC	III	835	(1) Erlotinib	–	12.7	50.8	67	^262^
				(2) Observation		12.4, *P* = 0.525	59.1, *P* = 0.903		
NCT00030446	OC/recurrent	II	50	Erlotinib + carboplatin	57	–	–	38	^429^
NCT00126542	OC/recurrent	II	13	Erlotinib + bevacizumab	15	4.1	11	–	^261^
NCT00130520	OC/advanced	II	40	Erlotinib + bevacizumab	23.1	4	–	30	^398^
NCT00059787	OC/advanced	II	56	Erlotinib + chemotherapy	29	34.3	–	35.71	^430^
NCT00217529	OC/advanced	I/II	159	Erlotinib + chemotherapy	Terminated because of gastrointestinal toxicity.				^431^
NCT00031993	CC/recurrent or persistent	II	28	Erlotinib	0	Only 1 patient PFS > 6 mths			^432^

*Unpublished data found in ClinicalTrials.gov

### Cetuximab

Cetuximab was demonstrated to have no additional benefit beyond chemotherapy in several phase II trials for CC.^244,245^ Moreover, in a phase II trial, the combination of cetuximab and topotecan induced a high rate of serious adverse reactions in the treatment of advanced CC.^246^ Another randomized phase II trial, MITO CERV-2 (NCT00997009), studied the efficacy of cetuximab plus carboplatin and paclitaxel in advanced or recurrent CC, showing no significant improvement in either the median PFS or the median OS.^247^ For OC, a phase II trial (NCT00086892) demonstrated modest activity of cetuximab in combination with carboplatin in patients with platinum-sensitive recurrent OC with an ORR of 32.1% and an increased incidence of hypersensitivity reactions.^248^ There is limited information about the clinical efficacy of cetuximab in EC.

### Trastuzumab

Trastuzumab treatment revealed no responses in a phase II trial with HER2-positive EC (NCT00006089).^249^ However, another randomized phase II trial (NCT01367002) of paclitaxel and carboplatin with or without trastuzumab in primary stage III or IV or recurrent HER2-positive uterine serous carcinomas showed an improvement in the median PFS in the trastuzumab combination arm (4.6 months longer, *P* = 0.005). In the population with primary advanced-stage disease, the median PFS was 17.9 months in the trastuzumab combination arm versus 9.3 months in the chemotherapy alone arm. In the population with recurrent disease, the median PFS was 9.2 versus 6 months, respectively.^250^ For patients with HER2 overexpression OC, trastuzumab showed modest activity with an ORR of 7.3% in a phase II trial.^239^ A clinical study in china demonstrated that the combination of abraxane and trastuzumab might have promising efficacy and adverse reaction in the treatment of recurrent OC, showing a control rate of 86.4%.^251^ However, there is limited information about the clinical efficacy of trastuzumab for CC.

### Pertuzumab

A randomized phase II trial (NCT00096993) of chemotherapy (gemcitabine) with or without pertuzumab in patients with platinum-resistant OC demonstrated an increased ORR in the pertuzumab combination arm.^252^ Furthermore, a phase III trial, PENELOPE (NCT01684878), evaluated the addition of pertuzumab to chemotherapy in patients with platinum-resistant OC with low tumor HER3 mRNA expression. However, the differences in the median PFS and OS were not statistically significant.^253,254^ In unselected patients with platinum-sensitive recurrent OC, a phase II trial (NCT02004093) showed that the addition of pertuzumab to carboplatin-based chemotherapy did not substantially prolong PFS.^255^ Also, there is limited information about the clinical efficacy of pertuzumab for EC and CC.

### TKIs

In clinical trials of small-molecule inhibitors, a phase II trial (NCT00096447) tested the efficacy of lapatinib and explored biological characteristics in persistent or recurrent EC.^256^ The analysis demonstrated that lapatinib had limited activity in unselected cases in EC, as well as in OC and CC.^257–260^ A phase II trial assessed the activity and tolerability of the combination of bevacizumab and erlotinib in recurrent OC with an ORR of 15%.^261^ Furthermore, a phase III trial (NCT00263822), evaluating the efficacy of maintenance erlotinib in OC patients after first-line chemotherapy, showed no improvement in PFS or OS.^262^ Moreover, this study failed to show a consistent correlation between EGFR mutational status/protein expression and clinical outcomes. For CC, a phase II trial evaluated the efficacy of erlotinib combined with chemoradiation in treating patients with locally advanced CC, showing a promising activity with a complete response of 94.4%.^263^ Other HER-targeted TKIs (e.g., gefitinib, canertinib, and vandetanib) showed minimal clinical activities in gynecological cancers in current clinical trials.^242,264–267^

Even though the present clinical evidences are not very satisfying, HER-targeted therapies continue to be investigated in gynecological cancers for their potent value for biomarker-selected patients (e.g., NCT01388621, NCT01367002, NCT02039791, NCT00292955, NCT03469531, NCT00317772, NCT01953926). Furthermore, preclinical data suggested the potential of novel combination strategies involving HER-targeted therapy, which are also investigated in ongoing clinical trials.^227,268–270^

### Other molecular targeted therapies

#### Ras/Raf/MEK

**Fig. 4:**
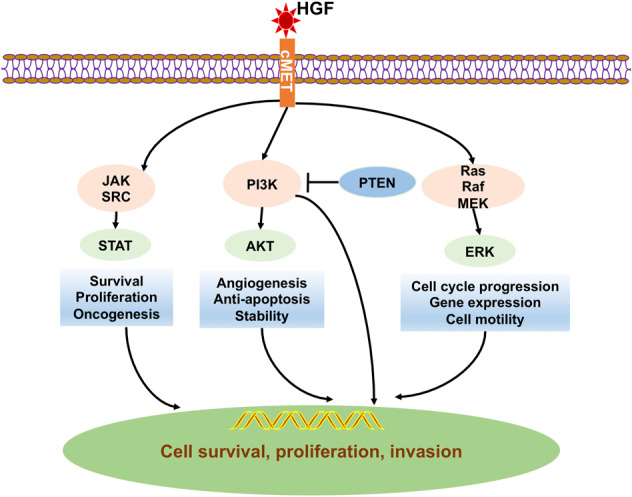
The HGF/c-MET signal transduction pathway and therapeutic interventions

In the Ras/Raf/MEK signaling pathway, Ras activation is the first process in activation of the mitogen-activated protein kinases (MAPKs) cascade.^271^ Upon Ras activation, Raf is recruited to the cell membrane where subsequent changes in Raf phosphorylation status result in activating MEK kinases (MEK1 and MEK2).^272^ MEK1 and MEK2 furtherly trigger Erk1 and Erk2. Finally, Erks regulate the activity of several transcription factors that induce the expression of multiple genes required for important cell activities (Fig. 4).^273^ The dysregulation of this pathway exists in many human tumors, making it an attractive antitumor target. Intensive preclinical researches have led to identifying Raf kinase inhibitors, as well as inhibitors of its downstream effector MEK kinase.^274–276^ The results of the completed phase II trials, which evaluated the efficacy of the Ras/Raf/MEK pathway inhibitors in treating gynecological cancers, are summarized in Table 9.

**Table 9:** Phase II trials (with results) of molecular targets in gynecological cancers

ID	Cancer/condition	No.	Target	Intervention	ORR (%)	mPFS (mon.)	mOS (mon.)	SAEs (%)	Refs
NCT01936363	OC	63	MEK	(1) Pimasertib + XL765	12.5	9.99	–	50	*
				(2) Pimasertib + placebo	12.1	12.71		56.25	
NCT00551070	OC/recurrent, low-grade serous	52		Selumetinib	15	–	–	63.46	^298^
NCT01011933	EC/recurrent or persistent	54		Selumetinib	6	2.3	8.5	64	^297^
NCT02538627	CC/recurrent or persistent	35		Trametinib + Uprosertib	7.1	3.6	14.8	57	^202^
NCT01935973	EC/ recurrent or persistent	26		Trametinib + Uprosertib	8.3	PFS at 6 months = 14%		61	^203^
NCT01047891 TRIAS	OC/platinum-resistant recurrent	185	Raf	(1) Sorafenib + topotecan	31	6.7	17.1	59	^290^
				(2) Placebo + topotecan	12	4.4, *P* = 0.0018	10.1, *P* = 0.017	51	
NCT00390611	OC/first-line treatment	85		(1) Sorafenib + PC	69	15.4	36.5	27.91	^288^
				(2) PC	74	16.3, *P* = 0.38	Not reached	23.81	
NCT00096200	OC/platinum-sensitive recurrent	36		(1) Sorafenib + PC	61	16.8	25.9	21.43	^291^
				(2) Sorafenib	15	5.6, *P* = 0.012	25.6, *P* = 0.974	16.67	
NCT00791778	OC/maintenance	246		(1) Sorafenib	–	12.7	–	21.14	^289^
				(2) Placebo		15.7		20.33	
NCT00093626	OC/third-line therapy	11		Sorafenib	–	2.00	11.78	low	^286^
NCT00436215	OC/recurrent	55		Sorafenib + bevacizumab	19	6.1		45.45	*
NCT00281515	OC/stage IIb–IV	105	Ras	(1) Lonafarnib + PC	–	11.5	20.6	–	^278^
				(2) PC		16.4, *P* = 0.0141	43.4, *P* = 0.012		
NCT01164995 M10MKO	OC/p53 mutated refractory	21	Wee1	Adavosertibc (AZD1775)	43	5.3	12.6	–	^341^
NCT01039207	OC/recurrent or persistent	31	c-MET	Rilotumumab	3.2	PFS at 6 months = 6.5%		45.16	^322^
NCT01716715	OC/recurrent	111		(1) Cabozantinib	7	5.3	19.4	–	^324^
				(2) Paclitaxel	24.1	5.5	Not reached		
NCT02315430 NRG-GY001	OC/recurrent	13		Cabozantinib	0	3.6	8.1	–	^323^
NCT00940225	OC	70		Cabozantinib	15	4.9	–	74.5	^433^
NCT02059265	OC/ recurrent or persistent	35	Src	Dasatinib	3.6	2.1	17.7	57.14	^331^
NCT01196741	OC/platinum-resistant recurrent	107		(1) Saracatinib + placitaxel	29	4.7	–	57.97	^434^
				(2) Placebo + placitaxel	43	5.3, *P* = 0.99		51.43	
NCT01175343	OC/platinum-resistant recurrent	45	Notch	RO4929097	0	1.3	–	22.73	^339^

*Unpublished data found in ClinicalTrials.gov

Lonafarnib is an orally protein farnesyltransferase inhibitor for H-ras, K-ras-4B, and N-ras.^277^ The addition of lonafarnib into first-line chemotherapy was investigated in a phase II trial (NCT00281515), and no effect was observed on prolonging PFS or OS in advanced OC.^278^ Sorafenib is a non-selective oral multikinase inhibitor with effects on angiogenesis through inhibition of the VEGF receptor.^279,280^ In addition, the antitumor effect of sorafenib is thought to be mediated through its inhibition of the Ras/Raf/MEK pathway, which is also frequently activated in advanced OC.^281,282^ It has been evaluated in more than 100 clinical trials in different cancer types, especially in large phase III studies in renal and liver cancers.^283–285^ It has been approved by the FDA for the treatment of renal, thyroid, and hepatocellular carcinoma. In OC, sorafenib showed antitumor activity in xenograft models and clinical studies.^286,287^ However, the results from a phase II trial (NCT00390611) in first-line treatment and maintenance therapy of OC showed no effect on prolonging PFS in sorafenib combination arm versus chemotherapy alone.^288^ The similar results were reported in patients with OC in complete remission (NCT00791778).^289^ On the other hand, a phase II trial (NCT01047891) demonstrated that sorafenib combined with topotecan as maintenance therapy significantly improved in the median PFS (2.3 months longer, *P* = 0.0018) and OS (7.1 months longer, *P* = 0.017) in patients with platinum-resistant recurrent OC.^290^ In patients with platinum-sensitive recurrent OC, sorafenib in combination with carboplatin and paclitaxel was reported to show promising activity with an ORR of 61%.^291^ Sorafenib was tested in early-phase clinical trial for CC patients receiving concurrent chemoradiation.^292^ However, it ended with early closure.

Selumetinib is an oral selective inhibitor of MEK1 and MEK2. It has shown activity against several advanced cancers.^293,294^ Since mutational alterations were found in the MAPK pathway in OC and EC, selumetinib-related clinical trials were conducted in gynecological cancers.^281,295,296^ For EC, selumetinib is well-tolerated in patients with recurrent or persistent disease, but with limited single-agent activity with an ORR of 6% (NCT01011933).^297^ A phase II trial (NCT00551070) demonstrated the potential activity of selumetinib in the treatment of recurrent low-grade OC with an ORR of 15%. It was suggested that inhibitors of the MAPK pathway should be further investigated in OC patients.^298^ Subsequently, an ongoing randomized phase II/III trial (NCT02101788) continues to study how well trametinib (another MEK inhibitor) works and compares it to the standard treatment in treating patients with low-grade OC. Trametinib, combined with GSK2141795 (an AKT inhibitor), has previously been tested in phase I and II studies.^299^ However, phase II clinical trials assessing this combination in EC (NCT01935973) or OC (NCT02538627) showed no clinical benefit.^202,203^ A phase II trial tested the combination of trametinib and GSK2141795 in recurrent CC with no confirmed response and a SD of 57%.^202^

In summary, while a powerful preclinical rationale suggests that inhibition of Ras/Raf/MEK signaling has promising potent as an antitumor targeted therapy, the clinical efficacy of this strategy in gynecological cancers is currently limited.

#### JAK/STAT

The janus kinase/signal transducer and activator of the tran-ions (JAK/STAT) pathway has been proved to mediate the action of cytokines, interferons and growth factors, and their control of gene expression.^300^ Activation of the JAK/STAT pathway and overexpression of STAT have been seen in many malignancies such as colorectal and breast cancers.^301,302^ Therefore, the JAK/STAT pathway is being focused as a potential target in cancer therapies. Ruxolitinib is an FDA-approved drug of JAK for treatment of patients with polycythemia vera.^303^ Preclinical studies demonstrated that ruxolitinib reduced OC cell viability.^304,305^ It enhanced the sensitivity of OC cells to other anticancer agents, and suppressed ovarian tumor growth in mice.^306,307^ These results supported the clinical investigation of ruxolitinib in OC patients. A phase I/II trial (NCT02713386) is trying to explore the effect of ruxolitinib phosphate when given together with paclitaxel and carboplatin in treating patients with stage III–IV OC.

#### HGF/c-MET

Tyrosine kinase receptor c-MET (cellular–mesenchymal to epithelial transition factor) is activated by hepatocyte growth factor (HGF) and it can trigger important cellular processes.^308^ Upon binding by HGF, MET is dimerized and activates cellular processes through the Ras/Raf/MEK and PI3K/AKT/mTOR pathways (Fig. 4).^309,310^ In a limited number of tumors, MET genetic lesions or mutations lead to the constitutive activation of MET.^311,312^ However, in a majority of malignancies, aberrant MET signaling derives from the upregulation of HGF transcription, leading to receptor and ligand overexpression.^313–315^

Since the publications of pioneer studies, the HGF/c-MET system has gained growing attention with its role in the pathogenesis of gynecological cancers.^316,317^ In a study analyzing 1115 advanced cancer patients, MET amplification was detected in 2.6% patients with solid tumors.^318^ But in OC, MET overexpression was detected in more than 20% (range from 22% to 41%) ovarian clear cell adenocarcinomas.^319,320^ And increased expression of HGF and c-Met signaling is associated with a poor prognosis of EC patients.^321^ Therefore, targeting the interaction of c-MET and HGF would be beneficial in treating gynecological cancers. Despite there are massive preclinical data on the HGF/c-MET axis, currently there is no FDA-approved indication of this targeted therapy in cancers.

The most tested drugs in HGF/c-MET axis are those blocking c-MET activity. Rilotumumab and cabozantinib are the most-studied c-MET inhibitors in gynecological cancers. The results of the completed clinical trials (phase II) investigating the safety and efficacy of them in gynecological cancers are summarized in Table 9. A phase II trial (NCT01039207) evaluated the rilotumumab in the treatment of persistent or recurrent OC. Only 1/31 achieved objective response, and only two patients got 6-month PFS.^322^ A phase II trial (NCT02315430) evaluated cabozantinib in treating patient with recurrent clear cell OC with no response.^323^ Another phase II trial (NCT01716715) compared cabozantinib versus weekly paclitaxel in treatment of persistent OC, with even worse OS and ORR in cabozantinib arm.^324^ These results do not warrant further evaluation of rilotumumab or cabozantinib as a single agent in targeted therapy of OC. There is currently limited information of the clinical efficacy of these agents in EC and CC.

#### Src

Sarcoma proto-oncogene tyrosine kinase (Src) is a downstream component of many growth factor receptors, such as VEGFR, EGFR, and c-MET.^325^ Src is thought to increase chemotherapy resistance through activating Ras and AKT.^326^ Preclinical studies showed that inhibiting Src resulted in enhancing apoptosis caused by cytotoxic drugs, such as paclitaxel, carboplatin, and gemcitabine.^327,328^ Src has been found to be overexpressed in gynecological cancers and promote resistance against chemotherapy.^328,329^ Dasatinib and saracatinib are the most-studied highly selective Src inhibitors in gynecological cancers.^330^ In a phase II trial (NCT01196741), it was reported that saracatinib did not improve activity of weekly paclitaxel in platinum-resistant OC.^331^ Another phase II trial (NCT02059265) showed that dasatinib had minimal activity as a single agent in patients with recurrent OC.^332^ Even though no obvious activity has been seen as a single agent, Src inhibitors used in combination with other antitumor agents are promising.

#### Notch

Notch signaling is a primordial, evolutionarily conserved cell-fate determination pathway that has great relevance to multiple aspects of cancer biology, from cancer stem cell to tumor immunity.^333,334^ Previous studies have shown that the Notch pathway is associated with the epithelial–mesenchymal transition (EMT) processes in OC and CC.^335–338^ Currently, several classes of Notch inhibitors have been developed, mainly composed of gamma-secretase inhibitors (GSIs), siRNA, and monoclonal antibodies against Notch pathways.^336^ RO4929097 is a GSI, which had insufficient activity as a single agent in platinum-resistant OC in a phase II clinical trial (NCT01175343).^339^

#### Cell cycle checkpoints

Wee1 is a kinase controlling G/M and S phase checkpoints via phosphorylation of the cyclin-dependent kinases. Ataxia-telangiectasia-mutated-and-Rad3-related kinase (ATR) plays an important role in the DNA damage response to replication stress, preventing the entry of cells with damaged DNA into mitosis (e.g., when the cancer cells are challenged by chemotherapy).^340^ These functions of Wee1 and ATR make them potential therapeutic targets. The activities of ATR inhibitors (e.g., AZD6738) and Wee1 inhibitors (e.g., AZD1775) have been investigated in early-phase trials in gynecological cancers^341^ (Tables 9 and 14).

#### Antibody–drug conjugates

Antibody–drug conjugates (ADCs) are complex engineered molecules composed of a monoclonal antibody conjugated to payload (e.g., cytotoxic drugs) via stable linkers.^342,343^ By binding to the antigens on the tumor cell surface, the ADCs release the drug components intracellularly and lead to the death of tumor cell. This site-selective drug delivery can reduce toxicities for patients by limiting the exposure of normal tissues to the cytotoxic drugs.^344^

Mirvetuximab soravtansine is an ADC for treatment of folate receptor α (FRα)-expressing tumors, comprising a humanized FRα-binding monoclonal antibody, a cytotoxic maytansinoid effector molecule DM4, and a cleavable disulfide linker.^345–347^ The FRα mediates the endocytotic uptake of folate, which has a role in amino acid, DNA and RNA metabolism as well as in methylation reactions.^348^ FRα is overexpressed in several cancers, including ovarian, lung, renal, endometrial, colorectal and breast cancers.^349^ Thus, it is a promising target for ADC design. The FRα expression in tumor is a response-predictive biomarker for patient selection. Preclinical studies showed it to have potent antitumor activities in OC xenografts.^350^ Phase I trials of mirvetuximab soravtansine in OC were conducted.^347,351^ In a population of patients with FRα-positive and platinum-resistant OC, mirvetuximab soravtansine showed an ORR of 26% and a median PFS of 4.8 months.^352^ However, the phase III FORWARD I trial (NCT02631876), comparing the safety and efficacy of mirvetuximab soravtansine to chemotherapy in platinum-resistant OC, was terminated because it did not meet prespecified primary endpoints. Another newly registered phase III trial (NCT04209855) is going to compare the efficacy chemotherapy in platinum-resistant OC with a high-level of FRα expression.

Tisotumab vedotin is a monomethyl auristatin E (MMAE) bearing ADC conjugated to an anti-tissue factor (TF) monoclonal antibody via a protease cleavable linker. TF is involved with tumor cell signaling and angiogenesis. Ongoing phase I/II trials GEN701/GEN702 (NCT02001623, NCT02552121), investigated tisotumab vedotin in solid tumor, including cervical, ovarian, endometrial, and other solid cancers. In the preliminary data released, 11/34 (32.4%) patients with CC achieved a response.^353,354^

Other ADCs continue to be investigated in a number of ongoing clinical trials (e.g., NCT03748186, NCT03835819, NCT01631552, NCT03657043, NCT03319628, NCT02988817, NCT02751918, NCT02606305, NCT02208375, NCT02996825).

#### Programmed death protein-1 pathway blockade

**Fig. 5:**
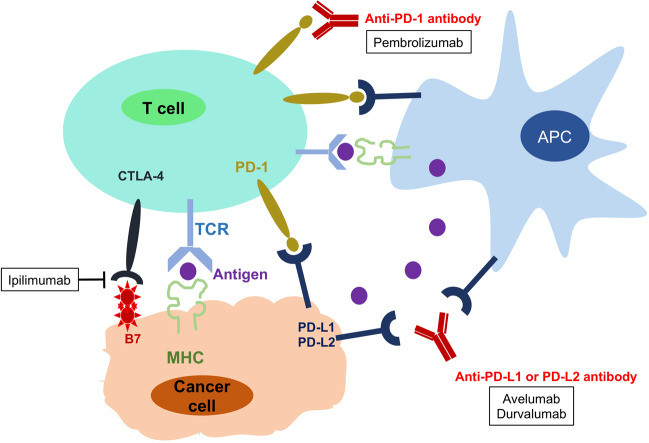
The immune checkpoint blockades. Antigen presenting cells (APC) take up antigen (Ag) released from tumor cells and present it to T cells. PD-1 receptors inhibit immune responses by engagement of PD-L1 and PD-L2. Therefore, monoclonal antibody blockading the PD-1 pathway results in enhancing antitumor immunity

Another class of novel alternative therapy in cancer treatment is the immunotherapeutic drug, particularly the agent that inhibits the immune checkpoint. Programmed death protein-1 (PD-1) is an immune checkpoint molecule which is more commonly studied in immunotherapy researches of gynecological cancers. It plays an important role in T-cell coinhibition and exhaustion, and subsequently helps tumor cells evade immune surveillance.^355^ Thus, monoclonal antibodies were developed as a promising cancer therapy targeting at blockading the PD-1 pathway in tumor progression. Although immune checkpoint inhibitors do not target to kill tumor cells directly, they play an antitumor role by enhancing T-cell functions (Fig. 5). The expression of immunosuppressive PD-1 ligands (PD-L1 or PD-L2) on the surface of tumor cells is an important predictive biomarker of response to PD-1 blockade.^356,357^ It is also indicated that mismatch repair-deficient (dMMR) tumors, including dMMR EC, are sensitive to PD-1 blockade.^358^ Anti-PD-1 agents (pembrolizumab and nivolumab) and anti-PD-L1 agents (atezolizumab, avelumab, and durvalumab) were FDA-approved drugs for several kinds of advanced-stage cancers, such as melanoma, NSLC and renal cell carcinoma.^359–361^ In 2017, pembrolizumab was approved by FDA for the treatment of patients with unresectable or metastatic solid tumors with a biomarker referred to as microsatellite instability-high (MSI-H) or dMMR.^362^ These biomarkers are most commonly found in colorectal, gastrointestinal, and endometrial cancers.^362–364^ Successively, pembrolizumab was approved in certain condition of CC and EC (Table 1), basing on findings from two phase II trials (KEYNOTE 158 and KEYNOTE 146).^365,366^ The results of the completed phase I/II trials of anti-PD-1/PD-L1 agents for ovarian, cervical, and endometrial cancers are summarized in Table 10. And other ongoing phase II/III trials investigating anti-PD-1/PD-L1 therapy (not in addition to other targeted agents) in gynecological cancers are listed in Tables 11 and 12.

**Table 10:** Completed phase I/II trials of anti-PD-1/PD-L1 in gynecological cancers

ID	Cancer/condition	Phase	No.	Intervention	ORR (%)	mPFS (mon.)	mOS (mon.)	SAEs (%)	Refs
–	OC/platinum-resistant recurrent	II	20	Nivolumab	15	3.5	20	40	^378^
NCT02873962	OC/recurrent	II	38	Nivolumab + bevacizumab	21	9.4	–	–	^377^
NCT00729664	OC/advanced	I	17	Nivolumab	5.9	–	–	5	^435^
NCT02488759 CheckMate 358 trial	CC/recurrent or metastatic	I/II	19	Nivolumab	26	–	21.9	–	^375^
NCT02257528	CC/persistent or recurrent	II	26	Nivolumab	4	–	–	24	^376^
NCT02674062 KEYNOTE100	OC/advanced or recurrent	II	376	Pembrolizumab	7.4–9.9	2.1	17.6	19.7	^373^
NCT02657889 KEYNOTE-162	OC/recurrent	I/II	62	Pembrolizumab+ niraparib	18	Not reached	–	–	^143^
NCT02537444	OC/recurrent	II	78	(1) ACP-196	2.9	–	–	21	*
KEYNOTE191				(2) ACP-196+ pembrolizumab	9.1			41	
NCT02628067 KEYNOTE 158	CC/advanced	II	98	Pembrolizumab	12.2	2.1	9	12.2	^365^
-	EC/dMMR recurrent or persistent	II	9	pembrolizumab	56	–	Not reached	0	^370^
NCT02501096 KEYNOTE 146	EC/advanced	II	54	Pembrolizumab+ lenvatinib	39.6	7.4	–	30	^366^
NCT02054806 KEYNOTE 028	EC/advanced, PD-L1( + )	Ib	24	Pembrolizumab	13	–	–	16.7	^367^
NCT02054806 KEYNOTE028	OC/advanced, PD-L1( + )	Ib	26	Pembrolizumab	11.5	1.9	13.8	3.8	^368^
	CC/advanced, PD-L1( + )		24		17	–	–	21	^369^
NCT02431559	OC/platinum-resistant recurrent	I/II	40	Durvalumab+ PLD	15	5.5	–	57.5	*
NCT01772004 JAVELIN Solid Tumor	OC/recurrent or refractory	Ib	124	Avelumab	9.7	2.7	10.8	6.5	^381^
NCT02912572	EC/MSS	II	33	Avelumab	27.6	–	–	19	^380^
	EC/POLE or MSI				6.25				
NCT01375842	EC/advanced or recurrent	Ia	15	Atezolizumab	13.3	1.7	9.6	13.3	^379^
NCT01375842	OC/recurrent	I	12	Atezolizumab	22.2	2.9	11.3	25.0	^436^
	EC/recurrent		15		13.3	1.4	9.6	43.3	

*dMMR* mismatch repair-deficient, *MSS* microsatellite stable, *MSI* microsatellite instable, *POLE* polymerase-ε. *Unpublished date found in clinicaltrials.gov

**Table 11:** Ongoing phase II trials of anti-PD-1/PD-L1 in gynecological cancers (not including novel combination therapy)

ID	Cancer/condition	No.	Start date	Intervention	Design	Status
NCT02725489	Women’s cancers	13	2016.6	Durvalumab	Non-randomized parallel	Not yet recruiting
NCT02811497 METADUR	OC/platinum-resistant recurrent	60	2016.9	Durvalumab + azacitidine	Single group	Recruiting
NCT03899610	OC/advanced	24	2019.7	Durvalumab + tremelimumab + chemotherapy	Single group	Recruiting
NCT03357757 LATENT	Virus associated cancer	39	2018.2	Avelumab + valproic acid	Single group	Recruiting
NCT03503786 MITO END-3	EC/advanced or recurrent	120	2018.4	Avelumab + PC vs. avelumab	Randomized parallel	Not yet recruiting
NCT02440425	OC/platinum-resistant recurrent	43	2015.8	Pembrolizumab + paclitaxel	Single group	Active, not recruiting
NCT02635360	CC/advanced	88	2016.1	Pembrolizumab maintenance/throughout, plus chemoradiation	Randomized parallel	Recruiting
NCT02608684 PemCiGem	OC/platinum-resistant recurrent	21	2016.2	Pembrolizumab + standard treatment	Single group	Active, not recruiting
NCT02530154	OC/stage III–IV	30	2016.7	Pembrolizumab + PC	Single group	Recruiting
NCT02899793	EC/recurrent or metastatic	25	2016.9	Pembrolizumab	Single group	Recruiting
NCT02865811	OC/platinum-resistant recurrent	26	2016.9	Pembrolizumab + doxorubicin	Single group	Active, not recruiting
NCT02901899	OC/recurrent	38	2016.11	Pembrolizumab + gemcitabine	Single group	Recruiting
NCT02900560	OC/platinum-resistant recurrent	34	2016.12	Pembrolizumab + azacytidine vs. pembrolizumab	Non-randomized parallel	Active, not recruiting
NCT02834975	OC/advanced	40	2016.12	Pembrolizumab + PC	Single group	Recruiting
NCT03192059 PRIMMO	CC or EC	43	2017.7	Pembrolizumab	Single group	Recruiting
NCT02549209	EC/recurrent	46	2017.8	Pembrolizumab + PC	Single group	Recruiting
NCT03126812	OC/stage IV	15	2017.11	Pembrolizumab as neoadjuvant	Single group	Recruiting
NCT03275506 NEOPEMBROV	OC/stage IV	45	2018.2	Pembrolizumab + chemotherapy vs. chemotherapy	Non-randomized parallel	Recruiting
NCT03029403	OC/advanced	42	2018.2	Pembrolizumab + DPX-Survivac (vaccine) + cyclophosphamide	Non-randomized parallel	Recruiting
NCT03410784 MITO28	OC/advanced	72	2018.4	Pembrolizumab + PC	Single group	Not yet recruiting
NCT03276013 TOPIC	EC/advanced, recurrent or metastatic	51	2018.5	Pembrolizumab + doxorubicin	Single group	Recruiting
NCT03539328 MITO27	OC/platinum-resistant recurrent	138	2018.6	Pembrolizumab + chemotherapy vs. chemotherapy	Randomized parallel	Not yet recruiting
NCT03732950	OC/recurrent	30	2019.3	Pembrolizumab	Single group	Recruiting
NCT03430700 PROMPT	OC/platinum-resistant recurrent	28	2019.5	Pembrolizumab + paclitaxel	Single group	Recruiting
NCT04375956	OC/platinum-resistant recurrent	100	2020.5	Pembrolizumab	Single group	Not yet recruiting
NCT04238988	CC/locally advanced	45	2020.3	Pembrolizuma + PC	Single group	Not yet recruiting
NCT03340376	CC/recurrent	48	2017.8	Atezolizumab vs. atezolizumab + doxorubicin vs. doxorubicin	Randomized parallel	Recruiting
NCT03612791	CC/advanced	190	2018.6	Atezolizumab + radiotherapy vs. radiotherapy	Randomized parallel	Recruiting
NCT03614949	CC/recurrent, persistent, or metastatic	26	2019.1	Atezolizumab	Single group	Recruiting
NCT02498600	OC/recurrent	96	2015.6	Nivolumab vs. nivolumab + ipilimumab	Randomized parallel	Active, not recruiting
NCT03241745	EC/metastatic or recurrent	40	2017.8	Nivolumab	Single group	Recruiting
NCT03808857	CC/recurrent or metastatic	80	2019.2	GB226	Single group	Recruiting
NCT03972722	CC/recurrent or metastatic	89	2019.5	GLS-010	Single group	Recruiting
NCT04188860	CC/recurrent	34	2019.12	Camrelizumab + paclitaxel	Single group	Recruiting
NCT04368273	CC/advanced	30	2020.5	Toripalimab	Single group	Not yet recruiting
NCT03104699	CC/advanced	211	2017.4	Balstilimab	Single group	Active, not recruiting

**Table 12:** Ongoing phase III trials of anti-PD-1/PD-L1 in gynecological cancers (not including novel combination therapy)

ID	Cancer/condition	No.	Start date	Intervention	Status
NCT02580058 JAVELIN Ovarian 200	OC/platinum-resistant, or- refractory recurrent	566	2015.12	Avelumab + PLD vs. avelumab vs. PLD	Active, not recruiting
NCT02891824 ATALANTE	OC/platinum-sensitive recurrent	405	2016.9	Atezolizumab vs. placebo, plus PC + bevacizumab	Recruiting
NCT03038100 IMagyn050	OC/stage III–IV	1300	2017.3	Atezolizumab vs. placebo, plus PC + bevacizumab	Active, not recruiting
NCT03353831	OC/platinum-resistant recurrent	664	2018.9	Atezolizumab vs. placebo, plus paclitaxel or PLD	Recruiting
NCT03556839	CC/stage IVb	404	2018.9	Atezolizumab vs. placebo, plus PC + bevacizumab	Recruiting
NCT03603184 AtTEnd	EC/advanced	550	2018.10	Atezolizumab vs. placebo, plus PC	Recruiting
NCT03635567 KEYNOTE-826	CC/persistent, recurrent, or metastatic	600	2018.10	Pembrolizumab vs. placebo, plus PC + bevacizumab	Recruiting
NCT03914612	EC/advanced or recurrent	810	2019.7	Pembrolizumab vs. placebo, plus PC	Recruiting
NCT04221945	CC/locally advanced	980	2020.4	Pembrolizumab vs. placebo, plus chemoradiation	Recruiting
NCT03830866 CALLA	CC/locally advanced	714	2019.2	Durvalumab vs. placebo, plus chemoradiation	Recruiting
NCT03981796 RUBY	EC/recurrent or stage III–IV	470	2019.7	Dostarlimab vs. placebo, plus PC	Recruiting
NCT03912415 FERMATA	CC/advanced	316	2019.9	Prolgolimab vs. placebo, plus PC + bevacizumab	Not yet recruiting

#### Anti-PD-1 agents

A phase Ib KEYNOTE-028 trial (NCT02054806) of pembrolizumab (10 mg/kg intravenously every 2 weeks) as a treatment of PD-L1–positive solid tumors showed that pembrolizumab was associated with a 17% ORR in CC cohort, a 13% ORR in EC cohort, and a 11.5% ORR in OC cohort, respectively.^367–369^ In KEYNOTE 158 trial (NCT02628067), pembrolizumab was investigated in a single cohort of recurrent or metastatic CC, resulting in an ORR of 12.2%. In the population of patients with PD-L1–positive tumors, the ORR was 14.6%. No response was observed in patients with PD-L1–negative tumors. The median OS was 9.4 months in the total population and 11 months in the PD-L1–positive tumor population.^365^ On the ground of this trial, the FDA-approved pembrolizumab for patients with recurrent or metastatic CC with disease progression on or after chemotherapy whose tumors expressed PD-L1, in 2018. As for EC, a phase II study evaluated the clinical efficacy of pembrolizumab in nine patients with recurrent or persistent EC with dMMR, and the results indicated that the ORR was 56%, the 12-month OS rate was 89%, and the median OS had not been reached.^370^ For EC patients without MSI or PD­-L1 expression status, another phase II KEYNOTE 146 trial (NCT02501096) assessed the activity and safety of lenvatinib plus pembrolizumab in patients with biomarker­-unselected advanced EC.^371^ Lenvatinib is an oral multikinase inhibitor targeting VEGFR, FGFR, PDGFR, RET, and KIT.^372^ An interim report of KEYNOTE 146 showed this combination of PD-1 blockade and inhibition of angiogenesis (as well as VEGF­-mediated immune suppression) was associated with antitumor activity with an ORR of 35.6%.^366^ In September 2019, the FDA granted accelerated approval to the combination of pembrolizumab and lenvatinib for the treatment of patients with advanced EC without MSI-H or dMMR and who have disease progression following prior systemic therapy, but were not candidates for curative surgery or radiation. For patient with recurrent OC, single-agent pembrolizumab showed modest activity in a phase II trial (NCT02674061) with an ORR of 7.4–9.9%.^373^ A phase I/II trial (NCT02657889) demonstrated that niraparib combined with pembrolizumab was tolerable and had promising antitumor activity for platinum-resistant current OC with an ORR of 18% and a disease control rate of 65%.^143^ Furthermore, a recent study identified two determinants of response to the combination of pembrolizumab and niraparib: the presence of mutational signature 3 as a surrogate of HRD and a positive immune score as a surrogate of interferon-primed, CD8-exhausted effector T cells in the tumor microenvironment. Presence of one or both tumor features was associated with significantly prolonged PFS while absence of both was associated with no response.^374^

Nivolumab is another well-known anti-PD-1 drug. As indicated by a phase I/II trial (NCT02488759), nivolumab had a promising activity in metastatic CC with an ORR of 26%.^375^ However, another phase II trial (NCT02257528) demonstrated that single-agent nivolumab exhibited low antitumor activity in recurrent CC with an ORR of 4% and a SD of 36%.^376^ In patients with platinum-resistant recurrent OC, early-phase trials showed that monotherapy of anti-PD-1 agents had promising activity.^377,378^

Dostarlimab (TSR-042) is an investigational humanized anti-PD-1 monoclonal antibody. It demonstrated robust clinical activity in patients with previously treated recurrent or advanced EC in both MSI-H and MSS subgroups. It is being evaluated in combination of bevacizumab and niraparib in patients with platinum-resistant OC (NCT03574779).

#### Anti-PD-L1 agents

In a phase Ia trial (NCT01375842) assessing atezolizumab (10 mg/kg intravenously every 3 weeks) in advanced/recurrent EC, the ORR was 13.3% (2/15) in all populations. Both these two patients were in population with PD-L1 status >5% of tumor-infiltrating immune cells (2/5). Moreover, a trend for higher PFS and OS was noticed with higher PD-L1 expression.^379^ A phase II trial (NCT02912572) of avelumab (10 mg/kg intravenously every 2 weeks) in patients with microsatellite stable (MSS), microsatellite instable (MSI), and POLE-mutated recurrent/persistent EC demonstrated an ORR of 6.25% in the MSS cohort and an ORR of 27.6% in the MSI/POLE cohort.^380^ As demonstrated in these clinical outcomes, PD-L1 status, dMMR, MSI, and POLE mutation were predictive biomarkers to identify the EC population who could benefit from PD-1 blockade. However, in patients with recurrent OC, a single-agent trial of anti-PD-L1 agents demonstrated only modest efficacy.^381^

Several clinical trials are further conducted to combine chemotherapy or other targeted therapies with anti-PD-1/PD-L1 agents in treatment of gynecological cancers. A phase II trials showed that the combination of durvalumab (10 mg/kg intravenously every 2 weeks) and doxorubicin was associated with an ORR of 15% in platinum-resistant recurrent OC. A great number of clinical trials have been designed and registered to investigate the efficacy and safety of anti-PD-1/PD-L1 agents combined with other targeted therapy in cancer treatment.

Currently, we find limited results from phase III trials investigating the safety and efficacy of anti-PD-1/PD-L1 agents in gynecological cancers. The reported interim results in OC are somehow disappointing. JAVELIN Ovarian 100 (NCT02718417), a phase III study of avelumab in combination with chemotherapy treating previously untreated OC patients, was terminated in 2018. The decision of termination was based on the results of a planned interim analysis that showed futility of efficacy. It was further reported that another ongoing phase III study of avelumab for platinum-resistant/refractory recurrent OC, JAVELIN Ovarian 200 (NCT02580058), did not meet prespecified primary endpoints of OS or PFS in patients. As of January 2020, there are dozens of ongoing phase III trials involving anti-PD-1/PD-L1 drugs in gynecological cancers, registered on ClinicalTrials.gov. The ongoing phase III trials are listed in Table 12.

Even though the preliminary results of phase III JAVELIN Ovarian trials are unsatisfying, anti-PD-1/PD-L1 drugs (either used as monotherapy or used in combination with chemotherapy, other immune checkpoint inhibitors, cancer vaccines or other targeted therapies) are still expected to be promising approaches, especially in the treatment of CC and EC.^382–384^

#### Selective estrogen receptor downregulators

In EC, type I (endometrioid histologies), the most common type, is associated with an excess estrogen exposure in the absence of counteractive effects of progesterone, mostly with expressing estrogen and/or progesterone receptors (ER/PR).^385–387^ Hormonal therapy is an alternative treatment to control metastatic or recurrent disease.^388,389^ In addition to the conventional progestin therapy, inhibition of estrogen-induced proliferation by anti-estrogenic agents has been evaluated in EC, including selective estrogen receptor modulators (SERMs) or downregulators (SERDs) and aromatase inhibitors.^390,391^

Fulvestrant, the main SERD, has an anti-proliferative effect through down regulation of ER and plays an antitumor role as both hormonal therapy and targeted therapy. Fulvestrant was approved by FDA for the treatment of postmenopausal metastatic ER/PR-positive breast cancer, not yet for gynecological cancers.^392^ A phase II trial (NCT00334295) evaluated the activity and toxicity of fulvestrant, in patients with advanced or recurrent ER/PR-positive EC.^393^ It demonstrated an ORR of 11.4% in the ITT group, with a median PFS of 2.3 months and a median OS of 13.2 months. However, another phase II trial showed minimal activity of fulvestrant in advanced, recurrent, or persistent EC.^394^ No patient demonstrated a complete or partial response in the 22 ER-negative patients, with a stable disease rate of 18% as the best response. The median PFS and OS were 2 and 3 months, respectively. In the 31 ER-positive patients, the ORR and stable disease rate were 16% and 29%, with a median PFS of 10 months and a median OS of 26 months, respectively. As for OC, fulvestrant was associated with a low ORR of 8% and a stable disease rate of 35% in ER-positive, multiply recurrent OC.^395^ The effect of anti-estrogenic agents in advanced or recurrent EC needs further investigations. Furthermore, combining hormonal therapy with targeted therapies is a novel strategy in treating certain gynecological cancers, which is being assessed in several ongoing clinical trials (e.g., NCT03643510, NCT03294694, NCT02730923, NCT02476955, and NCT02188550).

## Conclusion

From the large amount of clinical trials on targeted agents and molecular drugs, we can see the great enthusiasm in targeted therapies. Consequently, it has led to significant breakthrough in personalized medicine of antitumor treatment strategy, including gynecological cancers. According to current clinical evidence, PARP inhibitors have made a remarkable progress in treatment of OC depending on the identification of disease with HRD (e.g., BRCAm). As for EC, given the identification of hormone-dependent histological type and POLE/MSI molecular subtypes, the activity of PI3K/AKT/mTOR, PD-1, and hormone receptor-targeted therapies might be promising in treatment of patients with EC. Since CC is mostly associated with persistent infection of virus, immune-targeted therapies (e.g., anti-PD-1/PD-L1 agents) are expected to be prospective treatment strategy. For the future research, as we discussed previously, specific biomarkers are the keys to the tumor response of targeted drugs. Moreover, novel combination therapies, coinhibition of different targets, are worth conducting to overcome the drug resistance in cancer cells. A number of phase II/III clinical trials of novel combination strategies have been in progress (Tables 13 and 14).

**Table 13:** Ongoing phase III trials of novel combination targeted therapy in gynecological cancers

ID	Cancer/condition	No.	Start date	Target	Intervention	Status
NCT02502266 COCOS	OC/platinum-resistant or -refractory recurrent, BRCAm	680	2016.2	VEGF, PARP	Cediranib + olaparib vs. cediranib vs. chemotherapy	Recruiting
NCT02446600	OC/platinum-sensitive recurrent	549	2016.2	VEGF, PARP	Cediranib + olaparib vs. olaparib vs. chemotherapy	Active, not recruiting
NCT03522246 ATHENA	OC/stage III–IV	1012	2018.5	PARP, PD-1	Rucaparib + nivolumab vs. rucaparib + placebo vs. nivolumab + placebo vs. placebo	Recruiting
NCT03602859 ENGOT-0V44 /FIRST	OC/stage III–IV	912	2018.10	PARP, PD-1	Dostarlimab + niraparib vs. niraparib + placebo vs. placebo	Recruiting
NCT03884101 ENGOT-en9	EC/recurrent or stage III–IV	720	2019.4	VEGF, PD-1	Lenvatinib + pembrolizumab vs. chemotherapy	Recruiting
NCT03740165 KEYLYNK-001/ENGOT-ov43	OC/fist-line treatment	1086	2018.12	VEGF, PARP, PD-1	Pembrolizumab + olaparib vs. pembrolizumab + placebo vs. placebo, plus PC + bevacizumab	Recruiting
NCT03737643 DUO-O	OC/stage III–IV	1056	2019.1	VEGF, PARP, PD-1	Durvalumab + olaparib vs. durvalumab + placebo vs. placebo, plus PC + bevacizumab	Recruiting
NCT03806049 NSGO/AVANOVA-Triplet	OC/platinum-sensitive recurrent	337	2019.6	VEGF, PARP, PD-1	Niraparib + bevacizumab + dostarlimab vs. niraparib + bevacizumab vs. chemotherapy	Not yet recruiting

**Table 14:** Ongoing phase II trials of novel combination therapy in gynecological cancers

ID	Cancer/condition	No.	Started date	Targets	Drugs	Design	Status
NCT02345265	OC/recurrent	70	2015.12	VEGF, PARP	Cediranib + olaparib	Single group	Active, not recruiting
NCT02502266	OC/ platinum-resistant recurrent	680	2016.2	VEGF, PARP	Cediranib + olaparib vs. cediranib vs. olaparib	Randomized parallel	Recruiting
NCT02889900 CONCERTO	OC/platinum-resistant recurrent	62	2017.1	VEGF, PARP	Cediranib + olaparib	Single group	Active, not recruiting
NCT03117933 OCTOVA	OC/platinum-resistant recurrent	138	2017.3	VEGF, PARP	Paclitaxel vs. cediranib + paclitaxel vs. cediranib + olaparib	Randomized parallel	Active, not recruiting
NCT0331574 BARCCO	OC/recurrent	100	2017.6	VEGF, PARP	Paclitaxel vs. cediranib + olaparib	Randomized parallel	Recruiting
NCT03326193	OC/advanced	105	2018.1	VEGF, PARP	Niraparib + bevacizumab	Single group	Active, not recruiting
NCT03462212 MITO25	OC/advanced, high grade	234	2018.2	VEGF, PARP	Rucaparib + bevacizumab + chemotherapy vs. rucaparib + chemotherapy vs. bevacizumab + chemotherapy	Randomized parallel	Recruiting
NCT03570437 COPELIA	EC/advanced	129	2018.5	VEGF, PARP	Paclitaxel vs. cediranib + paclitaxel vs. cediranib + olaparib	Randomized parallel	Recruiting
NCT03476798	CC or EC/recurrent	70	2018.6	VEGF, PARP	Rucaparib + bevacizumab	Single group	Recruiting
NCT03660826	EC/recurrent, refractory, or metastatic	120	2018.9	VEGF, PARP	Cediranib vs. olaparib vs. cediranib + olaparib	Randomized parallel	Active, not recruiting
NCT03895788	OC/recurrent	24	2019.1	VEGF, PARP	Niraparib + brivanib	Single group	Recruiting
NCT02476798 Clovis-001	CC or EC/recurrent	70	2019.6	VEGF, PARP	Rucaparib + bevacizumab	Single group	Active, not recruiting
NCT04376073 ANNIE	OC/platinum-sensitive recurrent	40	2020.5	VEGF, PARP	Niraparib + anlotinib	Single group	Recruiting
NCT02921269	CC/recurrent	22	2017.3	VEGF, PD-1	Atezolizumab + bevacizumab	Single group	Not yet recruiting
NCT03572478	EC/metastatic or recurrent	60	2018.8	VEGF, PD-1	Rucaparib vs. nivolumab vs. rucaparib + nivolumab	Randomized parallel	Recruiting
NCT03526432	EC/advanced, recurrent or persistent	55	2018.8	VEGF, PD-1	Atezolizumab + bevacizumab	Single group	Recruiting
NCT03367871	CC/recurrent, persistent, or metastatic	39	2018.12	VEGF, PD-1	Pembrolizumab + bevacizumab	Single group	Recruiting
NCT03816553	CC/recurrent, persistent, or metastatic	49	2019.1	VEGF, PD-1	Camrelizumab + apatinib	Single group	Recruiting
NCT04068974	OC/platinum-resistant recurrent	28	2019.8	VEGF, PD-1	Camrelizumab + apatinib	Single group	Not yet recruiting
NCT04197219	EC/recurrent	26	2020.1	VEGF, PD-1	Pembrolizumab + axitinib	Single group	Not yet recruiting
NCT03797326	Advanced solid tumors	180	2019.2	VEGF, PD-1	Pembrolizumab + lenvatinib	Single group	Recruiting
NCT04236362	OC	30	2020.1	EGFR, PD-1	TQB2450 + anlotinib	Single group	Not yet recruiting
NCT02571725	OC/recurrent, BRCAm	50	2016.2	PARP, PD-1	Olaparib + tremelimumab	Single group	Recruiting
NCT02912572	EC/recurrent	70	2016.12	PARP, PD-1	Talazoparib + avelumab	Non-randomized parallel	Recruiting
NCT03330405 Javelin Parp Medley	OC/platinum-sensitive recurrent	242	2017.10	PARP, PD-1	Talazoparib + avelumab	Non-randomized parallel	Recruiting
NCT03572478	EC/ metastatic or recurrent	60	2018.8	PARP, PD-1	Rucaparib + nivolumab vs. nivolumab vs. rucaparib	Randomized parallel	Recruiting
NCT03651206 ROCSAN	OC/recurrent	196	2019.1	PARP, PD-1	Niraparib/dostarlimab + niraparib vs. chemotherapy	Randomized parallel	Active, not recruiting
NCT03824704	OC/HGSOC or endometroid	139	2019.5	PARP, PD-1	Rucaparib + nivolumab	Single group	Recruiting
NCT04068753 STAR	CC/platinum-resistant recurrent	150	2019.6	PARP, PD-1	Dostarlimab + niraparib	Single group	Active, not recruiting
NCT03951415 DOMEC	EC/recurrent	55	2019.7	PARP, PD-1	Durvalumab + olaparib	Single group	Recruiting
NCT03955471 MOONSTONE	OC/progressive or recurrent	68	2019.9	PARP, PD-1	Dostarlimab + niraparib	Single group	Recruiting
NCT04034927	OC/recurrent	170	2019.10	PARP, PD-1	Olaparib vs. olaparib + tremelimumab	Randomized parallel	Recruiting
NCT02953457	OC/recurrent or refractory, BRCAm	39	2017.6	PARP, PD-1, CTLA-4	Olaparib + durvalumab + tremelimumab	Single group	Recruiting
NCT02484404	Advanced solid tumors	384	2015.6	VEGF, PARP, PD-1	Olaparib + cediranib + durvalumab	Non-randomized parallel	Recruiting
NCT02873962	OC/recurrent	76	2016.11	VEGF, PARP, PD-1	Nivolumab + bevacizumab vs. nivolumab + bevacizumab+ rucaparib	Non-randomized sequential	Recruiting
NCT03574779 OPAL	OC/recurrent	41	2019.1	VEGF, PARP, PD-1,	Dostarlimab + niraparib + bevacizumab	Single group	Active, not recruiting
NCT04015739 BOLD	OC/recurrent	63	2019.2	VEGF, PARP, PD-1	MEDI4736 + olaparib+ bevacizumab	Single group	Recruiting
NCT03694262 EndoBARR	EC/persistent or progressive	30	2019.7	VEGF, PARP, PD-1	Atezolizumab + rucaparib + bevacizumab	Single group	Recruiting
NCT04361370 OPEB-01	OC/platinum-resistant recurrent	44	2020.4	VEGF, PARP, PD-1	Olaparib + pembrolizumab + bevacizumab	Single group	Active, not recruiting
NCT03699449 AMBITON	OC/platinum-resistant recurrent	68	2018.11	VEGF, PARP, PD-1, CTLA-4	Olaparib + cediranib vs. durvalumab + olaparib vs. durvalumab + chemotherapy vs. durvalumab + tremelimumab + chemotherapy	Randomized parallel	Recruiting
NCT02208375	EC or OC/recurrent	150	2014.11	PARP, mTOR, AKT	Olaparib + vistusertib vs. olaparib + capivasertib	Randomized parallel	Active, not recruiting
NCT03462342 CAPRI	OC/recurrent	86	2018.3	PARP, ATR	Olaparib + AZD6738	Single group	Recruiting
NCT04065269 ATARI	Gynecological cancers, AR1A loss	40	2019.11	PARP, ATR	AZD6738 vs. AZD6738 + olaparib	Randomized parallel	Recruiting
NCT04239014 DUETTE	OC/platinum-sensitive recurrent	192	2020.3	PARP, ATR	AZD6738 vs. AZD6738 + olaparib vs. placebo + olaparib	Randomized parallel	Not yet Recruiting
NCT03579316	OC/recurrent	70	2018.12	PARP, Wee	Adavosertib vs. adavosertib + olaparib	Randomized parallel	Recruiting
NCT03924245	OC/platinum-resistant recurrent	73	2019.12	PARP, HDAC	Olaparib + entinostat	Single group	Active, not recruiting
NCT02764333	OC/platinum-resistant recurrent	29	2016.5	PD-1, cancer vaccine	Durvalumab + TPIV200	Single group	Active, not recruiting
NCT03946358	CC/HPV +	47	2019.9	PD-1, cancer vaccine	Atezolizumab + UCPVax (vaccine)	Single group	Not yet Recruiting
NCT03015129	EC	80	2017.1	PD-1, CTLA-4	Durvalumab + tremelimumab vs. durvalumab	Randomized parallel	Recruiting
NCT03026062	OC/platinum-resistant recurrent	100	2017.5	PD-1, CTLA-4	Durvalumab vs. durvalumab + tremelimumab	Randomized parallel	Recruiting
NCT03277482	Gynecological cancer	32	2018.2	PD-1, CTLA-4	Durvalumab + tremelimumab + radiotherapy	Single group	Recruiting
NCT03355976	OC/advanced, recurrent, or metastatic	62	2018.4	PD-1, CTLA-4	Nivolumab + ipilimumab vs. nivolumab	Randomized parallel	Recruiting
NCT03894215	CC/recurrent	200	2019.3	PD-1, CTLA-4	Balstilimab + AGEN 1884	Randomized parallel	Recruiting
NCT02734004 TRU-D	OC/stage III–IV	24	2019.7	PD-1, CTLA-4	Durvalumab + tremelimumab	Single group	Recruiting
NCT04380805	CC/recurrent or metastatic	40	2020.5	PD-1, CTLA-4	AK104	Single group	Active, not recruiting
NCT03439085	CC/recurrent or metastatic	77	2018.11	PD-1, HPV vaccine	Durvalumab + MEDI0457	Single group	Recruiting
NCT04096911	CC	20	2019.7	PD-1, HPV vaccine	Sintilimab + quadrivalent HPV vaccine	Single group	Recruiting
NCT03835819	EC/advanced or recurrent	35	2019.9	PD-1, ADC	Pembrolizumab + mirvetuximab soravtansine	Single group	Recruiting
NCT03113487	OC/recurrent	28	2018.2	PD-1, p53	Pembrolizumab + p53MVA	Single group	Recruiting

*HDAC* histone deacetylase
